# Designing covalent organic frameworks with Co-O_4_ atomic sites for efficient CO_2_ photoreduction

**DOI:** 10.1038/s41467-023-36779-4

**Published:** 2023-02-28

**Authors:** Qian Zhang, Shuaiqi Gao, Yingying Guo, Huiyong Wang, Jishi Wei, Xiaofang Su, Hucheng Zhang, Zhimin Liu, Jianji Wang

**Affiliations:** 1 Key Laboratory of Green Chemical Media and Reactions (Ministry of Education), Collaborative Innovation Centre of Henan Province for Green Manufacturing of Fine Chemicals, School of Chemistry and Chemical Engineering, Henan Normal University, Xinxiang, Henan 453007 P. R. China; 2 Department of Electrical and Computer Engineering, National University of Singapore, Singapore, 117583 Singapore; 3 Beijing National Laboratory for Molecular Sciences, Key Laboratory of Colloid, Interface and Thermodynamics, CAS Research/Education Centre for Excellence in Molecular Sciences, Institute of Chemistry, Chinese Academy of Sciences, Beijing, 100190 P. R. China

**Keywords:** Photocatalysis, Metal-organic frameworks, Sustainability, Photochemistry

## Abstract

Cobalt coordinated covalent organic frameworks have attracted increasing interest in the field of CO_2_ photoreduction to CO, owing to their high electron affinity and predesigned structures. However, achieving high conversion efficiency is challenging since most Co related coordination environments facilitate fast recombination of photogenerated electron-hole pairs. Here, we design two kinds of Co-COF catalysts with oxygen coordinated Co atoms and find that after tuning of coordination environment, the reported Co framework catalyst with Co-O_4_ sites exhibits a high CO production rate of 18000 µmol g^−1^ h^−1^ with selectivity as high as 95.7% under visible light irradiation. From in/ex-situ spectral characterizations and theoretical calculations, it is revealed that the predesigned Co-O_4_ sites significantly facilitate the carrier migration in framework matrixes and inhibit the recombination of photogenerated electron-hole pairs in the photocatalytic process. This work opens a way for the design of high-performance catalysts for CO_2_ photoreduction.

## Abstract (web summary)

A class of inexpensive aminoanthraquinone organic dyes are shown to facilitate visible-light-driven CO_2_ reduction. Overall reaction efficiencies were found to be optimal when both electron donating and accepting groups were on a single dye molecule.

## Introduction

Over the past half century, the atmospheric concentration of CO_2_ has increased steadily because of the excessive consumption of fossil fuels and the continuous discharge of CO_2_, causing disastrous environmental problems, such as global warming and ecological deterioration^1,2^. As a result, CO_2_ neutralization technologies are urgently required to alleviate the pressures of both the existing high atmospheric CO_2_ level and the continuous increment in CO_2_ emissions^3,4^. A photocatalytic CO_2_ reduction reaction (CO_2_RR) has been considered an environmentally friendly solution for these problems, as light is clean, noninvasive, and remote-controllable. To date, the most used photocatalysts for the CO_2_RR are metal oxides^5,6^, sulfides^7^, polyoxometalates (POMs)^8^, metal-organic frameworks (MOFs)^9^, and conjugated microporous polymers^10^. Despite the promise of these photocatalysts, the production and selectivity of reaction products are quite low due to three essential limitations—poor light absorption efficiency, fast recombination of photogenerated electron-hole pairs, and less efficacious active sites—which often result in undesired side reactions^11^. Therefore, seeking more efficient photocatalysts is of great significance to overcome these obstacles.

Covalent organic framework (COF) is a class of organic polymers linked by reversible covalent bonds. COFs have highly periodic and modular crystalline structures, defining their programmable characteristics^12^. Recently, these porous frameworks have emerged as potential candidates for photocatalytic CO_2_RR to CO due to their large surface areas, astonishing π-conjugated skeletons, high chemical and thermal stabilities, tuneable pore sizes, and functionalities^13–15^. To enhance the photocatalytic activity of the parent COF, several strategies have been developed in recent years. Among these strategies, incorporating metal sites into the host frameworks of COF is a feasible and compelling method for improving the photocatalytic activity since it increases the number of active sites, facilitates electron transfer into the COF, and enhances the separation efficiency of photogenerated electron-hole pairs^15^. Nevertheless, when considering the chemical microenvironments integrated with metal and local binding modes^16^, different metal coordination sites, such as metal-N_4_^17^, metal-N_3_O^18^, metal-N_2_O_2_^10^, and metal-N_4_O^19^, often exhibit distinct performance characteristics for photocatalytic CO_2_RR. Unfortunately, the related structure-activity relationship is not clear, which results in a large deviation in the design of high-efficiency CO_2_ photoreduction catalysts. Therefore, the most significant consideration is to establish guidelines for a clear functional description of the metal-coordination centers, which is the core thought for understanding how to design a metal-COF catalyst with high efficiency^17^. In addition, the optimal coordination environments of metal centers in the COF matrixes are essential factors for catalyst design, as they determine the activities of metal sites^20^.

To this end, a programmable design approach, as a strategy for dividing complex systems into more manageable modules^21^, is usually used in the programmable chemical synthesis of reticular materials and offers a good opportunity to regulate the chemical microenvironments of metal-COF composites with specific purposes and rationalities^12,22^. Guided by this approach, metal-COF composites with modular structures are regarded as good platforms for CO_2_ photochemical conversion, which involves various rigid secondary units, such as organic functional groups and metal sites^12,23,24^. By means of the programmable approach, these basic structural modules may be selected and optimized to modulate the microenvironments for the catalysis^25^. However, using the programmable approach to design desired coordination modes in well-defined COF remains unexplored.

In this work, we design two types of cobalt Schiff base COF composites for the photocatalytic CO_2_RR to CO, in which cobalt is anchored by coordinated oxygen atoms. Benefiting from the controllable functional groups in the precursor modules and the strong coordination of cobalt (II)^26^, the cobalt-COF (Co-COF) was spontaneously formed according to the predesigned position of the -OH group in aldehyde modules by a programmable approach to provide precise coordination microenvironments. Through various spectral characterizations and theoretical calculations, a distinct Co-O_4_ coordination mode is identified. The corresponding Co-COF composite exhibits a remarkably high CO production rate of 18000 µmol g^−1^ h^−1^ and an excellent CO selectivity of 95.7%. The CO production rate is higher than and the selectivity is comparable to the current record reported for metal-COF photocatalysts^15,27,28^. Mechanistic investigations indicate that the supereminent photocatalytic performance of Co-COF with Co-O_4_ sites is mainly attributed to the strong separation ability of photoinduced electrons and holes although the contributions of high CO_2_ adsorption capacity and low charge-transfer resistance are also important.

## Results

### Construction and analysis of microenvironments

**Fig. 1:**
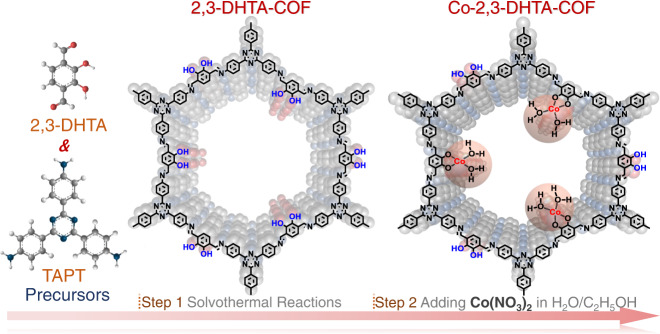
Schematic illustration of the synthesis of 2,3-DHTA-COF and Co-2,3-DHTA-COF as typical examples. In this study, TAPT works as the fixed module and 2,3-DHTA serves as the test module.

Here, 2,4,6-tris(4-aminophenyl)−1,3,5-triazine (TAPT) was used as a fixed module, and different aldehyde molecules were selected to serve as modules for building different porous frameworks containing isolated Co sites (Co-X-COF, where X represents aldehyde modules used to synthesize different COF matrixes, such as 2,3-dihydroxybenzene-1,4-dicarboxaldehyde (2,3-DHTA) and 2,4,6-trihydroxybenzene-1,3,5-tricarbaldehyde (TP)). Specifically, 2,3-DHTA was employed to form Co-O_4_ sites, while TP was applied to generate Co-O_3_N sites for comparative studies on different chemical environments. The structures of the parent COF and corresponding Co-COF are shown in Fig. 1 and Supplementary Fig. 1. For comparison, related characterizations for X-COF samples without Co sites were performed.

**Fig. 2:**
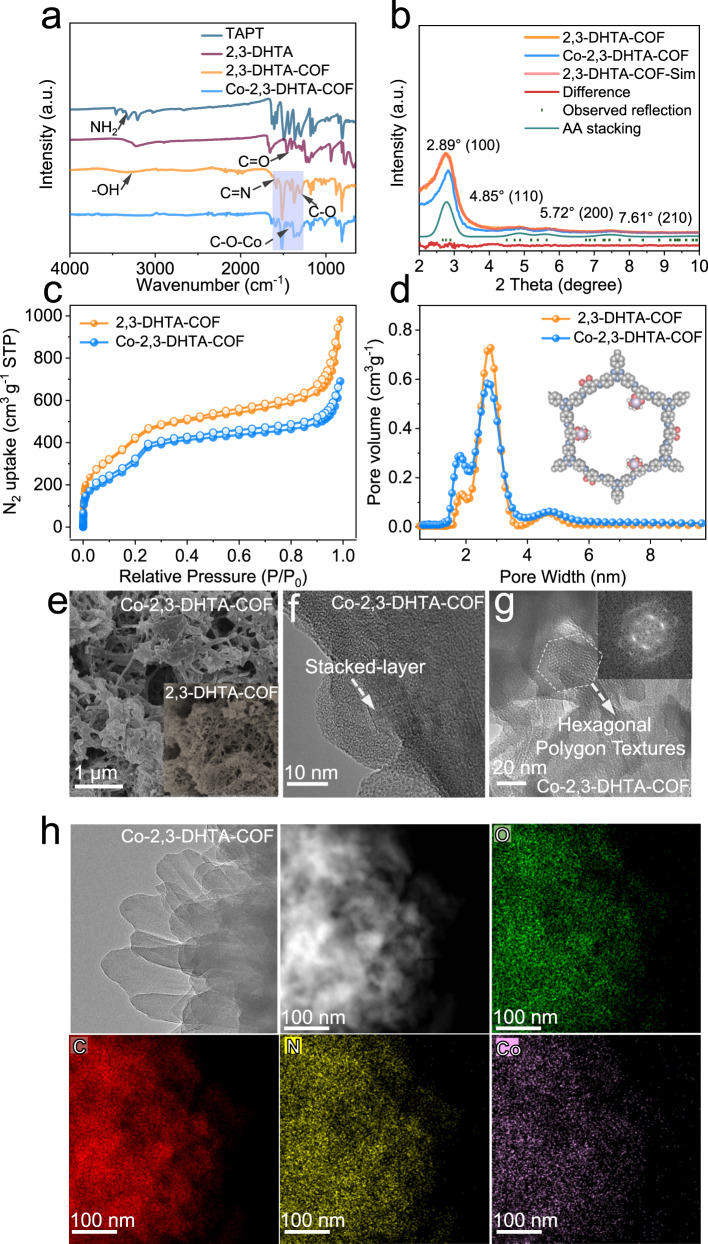
Spectral and structural comparison of 2,3-DHTA-COF and Co-2,3-DHTA-COF. **a** FTIR spectra. **b** Experimental and simulated X-ray diffraction (XRD) patterns. **c** N_2_ adsorption-desorption isotherms. **d** Pore size distribution calculated by the non-local density functional theory (NLDFT) model. **e** SEM images. **f**–**g** High-resolution TEM images. **h** Energy dispersive X-ray (EDX) maps of Co-2,3-DHTA-COF, with oxygen mapped in green, carbon mapped in red, nitrogen mapped in yellow, and cobalt mapped in purple. Scale bar: 100 nm.

Fourier transform infrared (FTIR) spectroscopy was employed to study the structural features of different frameworks. By using Co-2,3-DHTA-COF and its precursor modules as an example, the C = N stretching vibration peak was observed at 1623 cm^−1^, which refers to the formation of an imine bond between the 2,3-DHTA and TAPT modules, and accompanied by the vanishing of the amino band in the TAPT module (at approximately 3460, 3303, and 3209 cm^−1^) and the disappearance of the carbonyl band in the 2,3-DHTA module (at 1654 cm^−1^)^13,29^ (Fig. 2a and Supplementary Fig. 2a). This result indicated the successful construction of the 2,3-DHTA-COF matrix. After incorporating Co (II) into the COF matrix, the peak of C-O at 1291 cm^−1^, which was attributed to the stretching vibration of the phenoxy group in 2,3-DHTA modules^30^, was blueshifted to 1298 cm^−1^. At the same time, the new peaks at 1459 and 1347 cm^−1^ appeared for C-O-Co^31,32^ (Fig. 2a), providing direct evidence for the Co coordination environment. Likewise, the FTIR spectra of Co-TP-COF exhibited a similar trend in the change in the C-O vibration peak (Supplementary Fig. 2b). Solid-state ultraviolet-visible (UV-vis) diffuse reflection spectra showed that the absorption edges of Co-COF exhibited apparent redshifts relative to those of the parent COF matrixes (Supplementary Fig. 3), which was in line with the FTIR results and supported the formation of Co coordination centers.

The powder X-ray diffraction (PXRD) patterns of 2,3-DHTA-COF and Co-2,3-DHTA-COF are shown in Fig. 2b. A sharp diffraction peak at 2.89° appeared with three broad peaks at 4.85°, 5.72°, and 7.61°, corresponding to the (100), (110), (200) and (210) planes of 2,3-DHTA-COF^29^, respectively, which was in good agreement with the PXRD simulation result (Fig. 2b). However, no diffraction peaks of cobalt nanoparticles or cobalt salt were observed, suggesting that cobalt existed in the form of a coordination structure rather than in the form of cobalt salt or metallic cobalt nanoparticles in the COF matrix^10^. The same conclusion applied to Co-TP-COF (Supplementary Fig. 4). In addition, the PXRD simulation results indicated that the crystal structure was in AA stacking^29,33^. From the N_2_ adsorption isotherms (Fig. 2c, d), the incorporation of Co (II) into the parent COF decreased the surface area, but the pore size remained unchanged in the Co-COF. For example, the specific surface area was 1050 m^2^ g^−1^ for 2,3-DHTA-COF and 769 m^2^ g^−1^ for Co-2,3-DHTA-COF, while the pore size was 2.70 nm for both the COF and the Co-COF. A similar trend was observed for TP-COF and Co-TP-COF (Supplementary Fig. 5).

From the scanning electron microscopy (SEM) images (Fig. 2e and Supplementary Fig. 6), the COF and Co-COF showed predominantly ribbon-like morphologies^34^. In addition, the transmission electron microscopy (TEM) and high-resolution TEM images of Co-2,3-DHTA-COF (Fig. 2f, g and Supplementary Fig. 7) exhibited a stacked-layer structure with an interlayer spacing of 0.3 nm and hexagonal polygon pores with pore sizes of 2.6 nm. As the energy dispersive X-ray (EDS) maps suggested, cobalt (II) was homogeneously distributed throughout the COF matrix without any precipitation on the surface (Fig. 2h).

**Fig. 3:**
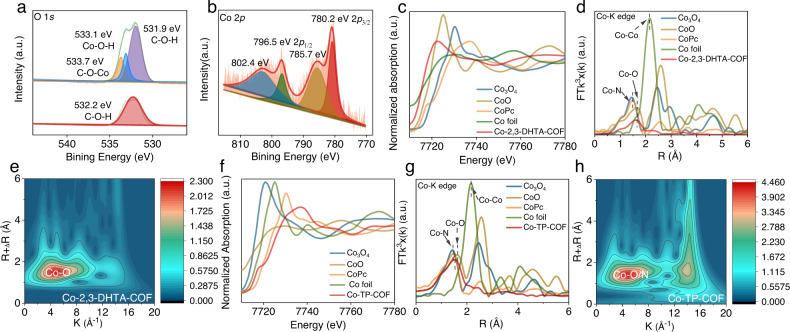
Coordination environment analysis. **a** High-resolution XPS spectra of O 1 *s* in Co-2,3-DHTA-COF and 2,3-DHTA-COF. **b** High-resolution XPS spectra of Co 2*p* in Co-2,3-DHTA-COF. **c**, **d** Normalized XANES spectra and FT-EXAFS spectra at the Co k-edge of Co-2,3-DHTA-COF, Co_3_O_4_, CoO, CoPc and Co foil samples. **e** WT-EXAFS spectrum of discriminating radial distance and k-space resolution of Co-2,3-DHTA-COF. **f** Normalized XANES spectra. **g** FT-EXAFS spectra. **h** WT-EXAFS spectrum for discriminating the radial distance and k-space resolution of Co-TP-COF.

The coordination environment of cobalt (II) in the 2,3-DHTA-COF matrix was investigated by X-ray photoelectron spectroscopy (XPS) (Supplementary Fig. 8). In detail, the O 1 *s* peak of Co-2,3-DHTA-COF was deconvoluted into three peaks (Fig. 3a): one at 531.9 eV for C-O-H^35^, one at 533.1 eV for Co-O-H and one at 533.7 eV for C-O-Co^10^. 2,3-DHTA-COF would exhibit a typical C-O-H peak at 532.2 eV^36^. Thus, the C-O-H peak shift (from 532.2 to 531.9 eV) and the new C-O-Co peak appearance (at 533.7 eV) suggested that the oxygen atom in Co-2,3-DHTA-COF was bonded with cobalt (II)^10^. A similar result was found for Co-TP-COF (Supplementary Fig. 9). In addition, no peak was observed for [NO_3_]^−^ in Co(NO_3_)_2_·6H_2_O at 407.0 eV^37,38^. Nevertheless, the N 1 *s* peak of Co-2,3-DHTA-COF was deconvoluted into two peaks (Supplementary Fig. 8e), one at 399.1 eV for C-N and the other at 401.1 eV for C = N^30^, and no N-O peak was found. These results indicated that nitrate was not present in the Co-COF. Therefore, the oxygen of [NO_3_]^−^ did not participate in the Co-O_4_ coordination. In addition, the Co 2*p*_3/2_ and Co 2*p*_1/2_ binding energies at 780.2 and 796.5 eV (Fig. 3b) illustrated the +2 oxidation state of Co in Co-2,3-DHTA-COF^39^, and the same conclusion was drawn from the XPS spectrum of Co-TP-COF (Supplementary Fig. 9). Inductively coupled plasma‒mass spectrometry (ICP‒MS) showed that the cobalt contents in Co-2,3-DHTA-COF and Co-TP-COF were approximately 2.14 and 1.05 wt.%, respectively, which was the maximum cobalt loading in the COF matrix. This high Co loading of Co-2,3-DHTA-COF indicated that the 2,3-DHTA module provided a more favorable coordination microenvironment for cobalt (II), which was consistent with the XPS analysis data (Supplementary Table 1). However, the cobalt contents in these two Co-COFs were much lower than the theoretical values (10.8 wt.% for Co-2,3-DHTA-COF and 9.4 wt.% for Co-TP-COF), suggesting that only a fraction of oxygen atoms in the Co-COF were bonded with cobalt (II)^10,30^.

A more accurate coordination environment of the cobalt species was disclosed by synchrotron-radiation-based X-ray absorption spectroscopy (XAS) analysis. X-ray absorption near-edge structure (XANES) spectra acquired for Co-2,3-DHTA-COF, Co-TP-COF, and the standard references (cobalt foil, CoO, CoPc (cobalt phthalocyanine), and Co_3_O_4_) are presented in Fig. 3c–e and Supplementary Figs. 10 and 11. For Co-2,3-DHTA-COF, the near-edge shoulder was at the same position as that for CoO, implying that the Co species were divalent in Co-2,3-DHTA-COF, which was in agreement with the result from XPS analysis. Moreover, the Fourier transformed extended X-ray absorption fine structure (FT-EXAFS) spectrum of Co-2,3-DHTA-COF in R space exhibited a dominant Co-O peak at 1.61 Å (Fig. 3d). This result was similar to the Co-O bond of the standard CoO in the range of 1.6−1.7 Å but different from the characteristic peak of the Co-Co bond (cobalt foil) at 2.16 Å or the Co-N bond (CoPc) at 1.45 Å. Accordingly, this result showed that the cobalt centers in the Co-2,3-DHTA-COF were surrounded by the oxygen atoms of the 2,3-DHTA aldehyde modules. More importantly, there were no other peaks of Co-2,3-DHTA-COF that were observed in the range of 2.3-3.0 Å, such as those of Co-O and Co-Co in the standard cobalt foil, CoO, and Co_3_O_4_ samples. Therefore, the above results corroborated that the cobalt-oxygen coordination structure was the only existing form of cobalt in Co-2,3-DHTA-COF.

The specific coordination number and hyperfine structure around the cobalt centers were investigated by fitting the EXAFS data for the first coordination shell of Co-2,3-DHTA-COF (Supplementary Fig. 10). Each cobalt atom was coordinated by four O atoms (Co-O_4_), and the average bond length of Co-O was 2.09 Å in the first shell (Supplementary Table 2). As shown in the wavelet transform (WT) contour plots of Co-2,3-DHTA-COF (Fig. 3e), the contour maximum was detected at 5 Å^−1^ (abscissa), and the main peak was at approximately 1.5 Å (ordinate), which corresponded to the Co-O coordination in the first shell. In addition, no maximum intensity of the Co-Co contribution was observed in the second shell at approximately 2.9 Å, providing additional evidence for the absence of the Co-Co bond in Co-2,3-DHTA-COF^26,40^. Relative to the coordination mode of Co (II) in Co-2,3-DHTA-COF, the peak position and intensity of Co-TP-COF in R space changed simultaneously, and the strong peak (1.53 Å) between the Co-N (1.45 Å) and Co-O (1.65 Å) peaks was confirmed to be Co-O_3_N. This difference in the cobalt (II) coordination mode between Co-2,3-DHTA-COF and Co-TP-COF fit well with the logic for microenvironment design via the regulation of the precursor aldehyde modules (Fig. 3f–h, Supplementary Fig. 10 and Table 2).

In order to determine whether the water molecules in Co(NO_3_)_2_·6H_2_O participated in Co-O coordination, thermogravimetric analysis (TGA) of the two Co-COFs was conducted. Interestingly, thermal peaks of crystalline water^41–43^ were observed at 217 and 249 °C from the first derivative of the TGA curves for Co-2,3-DHTA-COF and Co-TP-COF, respectively (Supplementary Fig. 12), suggesting that water molecules in Co(NO_3_)_2_·6H_2_O existed in the Co-COFs. Combined with the above results, the oxygen atoms of Co-O_4_ in Co-2,3-DHTA-COF clearly came from water molecules and the framework of 2,3-DHTA-COF because the coordination of [NO_3_]^−^ towards Co (II) was ruled out. To examine oxygen species from the framework in Co-O_4_, we conducted infrared and solid hydrogen−1 nuclear magnetic resonance (^1^H NMR) studies on Co-2,3-DHTA-COF and 2,3-DHTA-COF. Unfortunately, we failed to obtain any useful information due to the low content of Co (II) in Co-2,3-DHTA-COF and the low sensitivity of solid ^1^H NMR and infrared spectroscopy.

By considering the structural similarity of phenolic hydroxyl groups in 2,3-dihydroxybenzene−1,4-dicarboxaldehyde (one of the building blocks for the preparation of the Co-COF) to that in 2,3-DHTA-COF and the high sensitivity of liquid ^1^H NMR technology, we determined the ^1^H NMR spectra of 2,3-dihydroxybenzene-1,4-dicarboxaldehyde and Co-2,3-dihydroxybenzene-1,4-dicarboxaldehyde complex in d^6^-dimethyl sulfoxide (DMSO), where Co-2,3-dihydroxybenzene-1,4-dicarboxaldehyde complex was prepared under the same conditions as Co-2,3-DHTA-COF. Supplementary Fig. 13 showed that the peak at 10.72 ppm was ascribed to the phenolic hydroxyl protons of 2,3-dihydroxybenzene-1,4-dicarboxaldehyde, and the number of hydrogen atoms obtained by the area integral was 2. However, this peak weakened, and the number of hydrogen atoms was 1.6 in the Co-2,3-dihydroxybenzene−1,4-dicarboxaldehyde complex. This result indicated that a fraction of hydrogen in the phenolic hydroxyl groups of the Co-2,3-dihydroxybenzene−1,4-dicarboxaldehyde complex was lost to provide oxygen anions (deprotonated phenolic hydroxyl groups) in the presence of Co (II). Similar metal complexes in DMSO were also reported in the literature^42^.

From the above analyzes, the two adjacent oxygen anions in the COF frameworks were coordinated with Co (II). In this case, it was not possible for [NO_3_]^−^ to coordinate Co (II) in the Co-COF because of the requirement of electroneutrality. Again, this result showed that [NO_3_]^−^ was not involved in the Co-O_4_ coordination. Then, oxygen atoms from two H_2_O molecules were coordinated towards Co (II), which was supported by the thermal peaks of crystalline water observed for Co-2,3-DHTA-COF. Therefore, it was appropriate to state that the Co-O_4_ coordination mode was formed by two adjacent oxygen anions from Co-COF and two oxygen atoms from two H_2_O molecules, as shown in Fig. 1.

### Photocatalytic performance of CO_2_ to CO

In principle, when the bandgap of the COF was lower than the redox potential of the CO_2_RR, the COF can be used as the catalyst for the CO_2_RR. From the UV‒vis diffuse reflectance spectra of the Co-COF catalysts (Supplementary Fig. 3), the optical bandgaps of Co-2,3-DHTA-COF and Co-TP-COF were 1.60 and 2.17 eV, respectively (Supplementary Fig. 14). Mott-Schottky plots showed that these two Co-COFs were n-type semiconductors with typical positive slopes (Supplementary Fig. 15), and the conduction band minimum (CBM) edges of Co-2,3-DHTA-COF and Co-TP-COF at pH = 7 were calculated to be −0.67 and −0.84 V (vs. normal hydrogen electrode (NHE)), respectively (Supplementary Fig. 16a). Therefore, the CBM potentials (−0.67 and −0.84 V) of these two Co-COFs were lower than the redox potential of CO_2_ to CO (−0.53 V) but much higher than the lowest unoccupied molecular orbital energy level (−1.31 V vs. NHE) of the photosensitizer [Ru(bpy)_3_]Cl_2_. Thus, the photoinduced electrons transferred from the photosensitizer [Ru(bpy)_3_]Cl_2_ to Co-COF, and the CO_2_RR was thermodynamically feasible (Supplementary Fig. 16b).

**Fig. 4:**
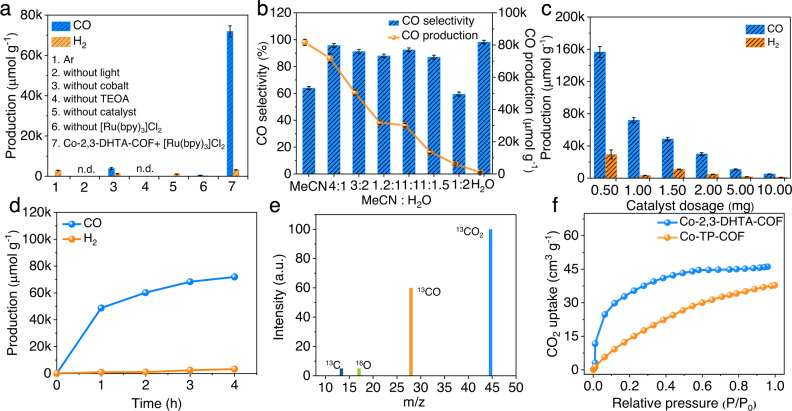
Optimization of the catalytic conditions using Co-2,3-DHTA-COF as the catalyst. **a** Control experiments of catalytic conditions. **b** Effect of the acetonitrile/H_2_O volume ratio on the photocatalytic CO_2_RR. **c** Dependence of catalyst mass loading on CO and H_2_ production within 4 h. **d** CO and H_2_ evolution with irradiation time for the CO_2_RR catalyzed by Co-2,3-DHTA-COF under visible light irradiation. **e** Gas chromatography‒mass spectrometry for ^13^C isotope tracer tests. **f** CO_2_ adsorption isotherms of the two Co-COFs at 25 °C. The error bars for the production and selectivity uncertainty of CO and H_2_ represent one standard deviation based on three independent samples.

Based on the above results, we performed photocatalytic CO_2_RR experiments in acetonitrile (MeCN)/water mixed solution under simulated visible light irradiation (λ ≥ 420 nm), with [Ru(bpy)_3_]Cl_2_ and triethanolamine (TEOA) as the potential photosensitizer and electron donor, respectively. Before estimating the CO_2_ photocatalytic performance of the Co-COF, a series of control experiments were performed to optimize the photocatalytic reaction conditions. The results are given in Fig. 4a–c. It was found from Fig. 4a and Supplementary Fig 17 that no CO was produced without visible light, TEOA or Co-2,3-DHTA-COF, and H_2_ was detected in the presence of TEOA and [Ru(bpy)_3_]Cl_2_ but no Co-2,3-DHTA-COF. Only in the presence of Co-2,3-DHTA-COF and TEOA, a small amount of CO and H_2_ were observed. However, when [Ru(bpy)_3_]Cl_2_ and Co-2,3-DHTA-COF simultaneously existed in the system, the CO production was greatly improved. These results indicated that [Ru(bpy)_3_]Cl_2_, Co-2,3-DHTA-COF, and TEOA worked as photosensitizers, photocatalysts and electron donors, respectively. Moreover, the effects of other factors, such as the ratio of acetonitrile/water and the photocatalyst amount, on photocatalytic activity and CO selectivity were explored to provide the best photocatalytic conditions. The optimized volume ratio of acetonitrile/water was clearly 4:1 (Fig. 4b), and 1.00 mg of catalyst loading was adopted to improve CO selectivity although the best production rate was observed at a dose of 0.50 mg (Fig. 4c and Supplementary Fig. 18). A similar catalyst dosage was often used in previous photocatalytic studies^10,13,40,44^. Under the optimized conditions, CO_2_RR to CO was performed, and the gaseous and liquid products in the photocatalytic reduction system were detected by gas chromatography and ^1^H NMR spectroscopy, respectively. H_2_ and CO were detected in the gaseous products, but no liquid product was detectable (Supplementary Figs. 19 and 20).

Figure 4d displays the variation in CO and H_2_ production with reaction time using the Co-2,3-DHTA-COF catalyst. The CO production was continuously produced in the initial 4 h, but the production rate significantly decreased after 1 h upon light illumination. This retardation effect was attributed to the degradation of the photosensitizer [Ru(bpy)_3_]Cl_2,_ as evaluated from the obvious decrease in its light absorption intensity (Supplementary Fig. 21). A similar phenomenon was reported in previous photocatalysis investigations^45–47^. Supplementary Fig. 22 displays the CO and H_2_ production from the CO_2_RR catalyzed by the Co-COF and the parent COF for 4 h under the same conditions. Notably, the optimal Co-2,3-DHTA-COF showed a very high CO production rate of 18,000 µmol g^−1^ h^−1^ with a H_2_ production rate of 800 µmol g^−1^ h^−1^ (Supplementary Tables 3 and 4). Thus, the selectivity towards the CO product was 95.7%, and the relevant turnover frequency (TOF) reached 111.8 h^−1^. The CO production rate of Co-2,3-DHTA-COF was 23.6 times that of 2,3-DHTA-COF. Compared with the COF-based catalysts reported in the literatures, the optimal Co-2,3-DHTA-COF offered a higher CO production rate than the current record reported in the photocatalytic CO_2_RR-to-CO as well as comparable CO selectivity (Supplementary Table 5). However, Co-TP-COF exhibited a lower production rate (11,600 µmol g^−1^ h^−1^) and selectivity (76.6%) for CO. To exclude the effects of the cobalt contents of the two Co-COFs on their catalytic performance levels, the Co-2,3-DHTA-COF sample with a cobalt content of 1.12 wt.%, which was similar to that in Co-TP-COF (1.05 wt.%), was prepared and applied for the photocatalytic CO_2_RR to CO. A CO production rate of 13,900 µmol g^−1^ h^−1^ and a selectivity of 90.4% were achieved, indicating that the different catalytic performance levels of Co-2,3-DHTA-COF and Co-TP-COF were mainly caused by the difference in Co coordination modes. Obviously, the Co-O_4_ site significantly improved the catalytic performance of Co-COF for the CO_2_RR to CO, which was in good agreement with our design goals.

The apparent quantum efficiency (AQE) of the CO_2_ photocatalytic process over Co-2,3-DHTA-COF was then studied at various wavelengths, and the variety of AQE values with wavelengths followed the characteristic absorption spectrum of [Ru(bpy)_3_]Cl_2_ (Supplementary Fig. 23). The highest AQE value was 0.47% at 450 nm (Supplementary Table 6). These results confirmed that [Ru(bpy)_3_]Cl_2_ functioned as the photosensitizer in this work. In addition, the produced ^13^CO (m/z = 29) detected by gas chromatography‒mass spectrometry (Fig. 4e) verified that the generated CO came from CO_2_ rather than the other organic molecules, such as the photosensitizer [Ru(bpy)_3_]Cl_2_, electron donor triethanolamine and COF matrix.

Encouraged by the above results, we evaluated the photocatalytic reduction performance of Co-2,3-DHTA-COF for low-concentration CO_2_. To this end, 10 vol.% CO_2_ (a typical CO_2_ concentration in the exhaust gas of coal-fired power plants) in N_2_ was taken for a 4 h conversion. The optimal Co-COF still exhibited an excellent CO production rate of 9600 µmol g^−1^ h^−1^ (Supplementary Fig. 24a), which even exceeded the values of many other metal-COF catalysts in a pure CO_2_ atmosphere (Supplementary Table 5). Although the selectivity of CO decreased and the production rate of H_2_ was more than twice that of CO, this H_2_-CO mixed gas was the so-called syngas that was an important raw material for the industrial synthesis of high value-added chemicals.

Moreover, natural sunlight irradiation was used for the photocatalytic CO_2_RR to CO under reaction conditions similar to those mentioned above. For this purpose, a 4-h photocatalytic reaction (from 10:0 am to 14:0 pm) per day was conducted for 3 consecutive days in December on outdoors of the campus at the location of east longitude 113°54'46” and north longitude 35°19'46”. Remarkably, on sunny days in winter, the average production rate of the photocatalytic CO_2_RR to CO catalyzed by Co-2,3-DHTA-COF reached 11000 ± 20 µmol g^−1^ h^−1^, and the selectivity towards CO reached 89% (Supplementary Fig. 24b). Considering the low temperature (approximately 19 °C) and weak light intensity (less than 50 mW cm^−2^) during testing (Supplementary Fig. 24c, d and Table 7), such a high CO production rate and selectivity indicated that with the advantages in the microenvironment of Co-2,3-DHTA-COF, natural sunlight was utilized for photocatalytic CO_2_RR to CO with effect, highlighting the high potential of the programmable approach in the design of specific microenvironments for practical applications.

In addition, to examine the stability of Co-2,3-DHTA-COF in CO_2_ photocatalytic reduction to CO, fresh [Ru(bpy)_3_]Cl_2_ was added to the system after each cycle to maintain a continuous process for the catalytic reaction. The good photocatalytic stability of Co-2,3-DHTA-COF was verified in three CO_2_ reduction cycle tests with a slight deterioration (Supplementary Fig. 25). Furthermore, the good chemical stability of Co-2,3-DHTA-COF was confirmed by comparing its TEM images, FTIR spectra, PXRD patterns, and XPS spectra before and after the catalytic process (Supplementary Figs. 26 and 27).

### Reaction mechanism

The high photocatalytic CO_2_ reduction performance of Co-2,3-DHTA-COF inspired us to investigate the CO_2_ photoreduction mechanism by isothermal CO_2_ adsorption, various ex/in situ spectral measurements, and density functional theory (DFT) calculations. Since CO_2_ adsorption was the first action before the catalytic steps, we first studied the adsorption isotherm of CO_2_ on the parent COF and Co-COF. The CO_2_ adsorption capacities of Co-2,3-DHTA-COF and Co-TP-COF were 46.1 and 37.8 cm^3^ g^−1^ (Fig. 4f and Supplementary Fig. 28), which were 3.8 times that of 2,3-DHTA-COF and 1.2 times that of TP-COF, respectively. The higher CO_2_ adsorption capacity of Co-2,3-DHTA-COF was attributed to its chemisorption for CO_2_ through the stronger coordination interaction of CO_2_ with Co (II) where the CO_2_ desorption peak was observed at ~212 °C, while the CO_2_ adsorption of 2,3-DHTA-COF was physical in nature^48,49^ since its CO_2_ desorption peak was observed at approximately 80 °C, as determined by a temperature-programmed CO_2_ desorption experiment (Supplementary Fig. 29). In addition, although the CO_2_ adsorption capacity of TP-COF (33.5 cm^3^ g^−1^) was significantly higher than that of 2,3-DHTA-COF (12.8 cm^3^ g^−1^), the CO_2_ adsorption capacity of Co-TP-COF was less than that of Co-2,3-DHTA-COF. Given that 2,3-DHTA-COF and TP-COF had the same topological structure, this result suggested that Co-O_4_ coordination in 2,3-DHTA-COF, rather than the topological structure, played a crucial role in the enhancement of CO_2_ adsorption capacity. Benefiting from the microenvironment constructed by the 2,3-DHTA modules, the optimal Co-2,3-DHTA-COF exhibited a high capacity CO_2_ adsorption, which was favorable for the CO_2_RR to CO.

It is known that upon illumination with visible light, the electrons in semiconducting materials could move to the conduction band to create holes in the valence band. The electron-hole separation efficacy was one of the most important factors for CO_2_ reduction. Thus, the photogenerated electron-hole separation efficiency was determined by photoluminescence (PL) spectroscopy. The steady-state PL spectra (Supplementary Fig. 30a) indicated that the strong emission of the photosensitizer [Ru(bpy)_3_]Cl_2_ was effectively quenched in the presence of Co-COF/COF, while the PL spectrum of [Ru(bpy)_3_]Cl_2_ was not quenched with various amounts of the sacrificial agent TEOA (Supplementary Fig. 31a), as reported previously^50^. However, with the addition of Co-2,3-DHTA-COF, the PL spectrum of the excited [Ru(bpy)_3_]Cl_2_ was gradually quenched, but there was almost no influence on the visible absorption spectrum of [Ru(bpy)_3_]Cl_2_ (Supplementary Fig. 31b, c). This result suggested that the PL spectrum directly quenched by Co-2,3-DHTA-COF in the photocatalytic system was not static quenching because static quenching, along with the formation of complexes between the luminophore and quencher, usually resulted in a change in the visible absorption spectrum of the luminophore^47,51^. In addition, the plot of PL intensity versus the content of Co-2,3-DHTA-COF (Supplementary Fig. 31d) turned out to have upwards curvature when fitted with the Stern−Volmer equation^52^, indicating that the PL of [Ru(bpy)_3_]Cl_2_ was not inherently collision quenching^47,52^. Therefore, the quenching of the PL intensity of [Ru(bpy)_3_]Cl_2_ was directly caused by the transfer of photoexcited electrons from [Ru(bpy)_3_]Cl_2_ to Co-2,3-DHTA-COF.

The evidence for this transfer was provided by ultrafast femtosecond time-resolved transient absorption (fs-TA) spectroscopy. As can be seen in Supplementary Fig. 32, after excitation of [Ru(bpy)_3_]Cl_2_, a bleaching peak was observed at approximately 450 nm, and the strength of this peak decreased with increasing delay time, which indicated that the recombination of a fraction of electrons and holes occurred in [Ru(bpy)_3_]Cl_2_ with the prolonged delay time. For Co-2,3-DHTA-COF, the strong and broad photoinduced bleaching peaks were observed at 490 nm and 550–650 nm (Supplementary Fig. 33a), which was attributed to the generation of photoexcited electrons in the conduction band of Co-2,3-DHTA-COF^53,54^. As the delay time increased to 1 ns, these peaks were not observed in Co-2,3-DHTA-COF due to the recombination of electrons and holes. Similar results were obtained for Co-TP-COF and 2,3-DHTA-COF (Supplementary Fig. 33b, c). Nevertheless, when Co-2,3-DHTA-COF was added into the solution of [Ru(bpy)_3_]Cl_2_, the peak strength of Co-2,3-DHTA-COF at approximately 550–650 nm increased with increasing delay time (Supplementary Fig. 32d), indicating that the electron transferred from [Ru(bpy)_3_]Cl_2_ to Co-2,3-DHTA-COF^27,55^. Therefore, the above results suggested that the photoelectrons were transferred from the excited state of [Ru(bpy)_3_]Cl_2_ to Co-COF/COF.

Compared with the COF matrixes, Co-COF displayed more significantly quenched photoluminescence emission and enhanced electron trapping and charge carrier separation. Thus, an adequate supply of electrons was realized by COF with abundant cobalt sites to optimize the elementary process of CO_2_ photocatalytic reduction. In addition, the significantly decreased lifetime of [Ru(bpy)_3_]Cl_2_ in the presence of Co-COF/COF also supported favorable electron transfer. Compared with Co-2,3-DHTA-COF, Co-TP-COF exhibited a less pronounced PL quenching effect and leaded to a longer lifetime of [Ru(bpy)_3_]Cl_2_ (173 ns in the presence of Co-TP-COF versus 156 ns in the presence of Co-2,3-DHTA-COF) (Supplementary Fig. 30b), which suggested that the recombination of photogenerated electron and hole pairs was better prevented by Co-2,3-DHTA-COF, and long-lived electrons were provided for CO_2_ reduction. For Co-TP-COF and 2,3-DHTA-COF, when [Ru(bpy)_3_]Cl_2_ was added, there was no obvious change in the fs-TA spectra within 7 ns (Supplementary Fig. 33e, f), indicating the rapid recombination of electrons and holes^56,57^. This result further verified that Co-2,3-DHTA-COF induced a high separation efficiency of electron and hole pairs for excited [Ru(bpy)_3_]Cl_2_. Furthermore, the lifetimes of the photogenerated electrons in Co-TP-COF and Co-2,3-DHTA-COF were evaluated by PL decay spectroscopy. By fitting the curves in Supplementary Fig. 34 with a biexponential equation^54^, the average lifetime (τ_avg_) values of photogenerated electrons in Co-2,3-DHTA-COF and Co-TP-COF were calculated to be 3.17 and 2.12 ns, respectively, supporting the result from the steady-state PL measurement. As such, the isolated Co metal sites anchored on 2,3-DHTA-COF facilitated photoinduced electron separation, resulting in enhanced CO_2_ photoreduction efficiency.

Next, the surface charge transfer efficiency was measured by electrochemical impedance spectroscopy (EIS) (Supplementary Fig. 35), and the charge transfer resistance simulated by the equivalent circuit followed the sequence Co-2,3-DHTA-COF (23 Ω) < Co-TP-COF (42 Ω) (Supplementary Table 8). Considering that the Co content in 2,3-DHTA-COF was higher than that in Co-TP-COF, this order suggested an inverse relation between interfacial charge transfer resistance and the contents of cobalt sites. Similarly, the Co-COF samples showed lower charge transfer resistance levels than their parent COF matrices. These results were well supported by the transient photocurrent response, where the transient photocurrent density of Co-2,3-DHTA-COF was significantly higher than that of Co-TP-COF, indicating a relatively high charge migration rate (Supplementary Fig. 36). Obviously, cobalt-active centers with different microenvironments played crucial roles in the CO_2_ adsorption, electron transportation, and separation of electrons and holes. Therefore, the Co-O_4_ sites in Co-2,3-DHTA-COF clearly enabled this Co-COF to provide the high CO_2_ adsorption capacity, low charge-transfer resistance, and strong separation of electrons and holes, resulting in high photocatalytic performance of the CO_2_RR to CO.

**Fig. 5:**
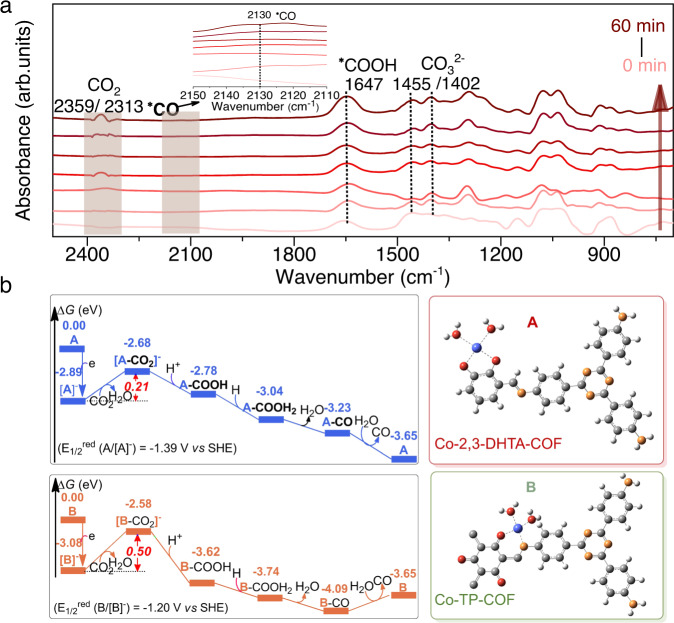
In situ ATR-FTIR spectra and reaction Gibbs free energy change. **a** Room temperature in situ ATR-FTIR spectra of the Co-2,3-DHTA-COF system to detect the formation of intermediates during photoreduction. **b** Gibbs energy profiles of the CO_2_RR to CO over Co-2,3-DHTA-COF (A, blue line) and Co-TP-COF (B, orange line).

Moreover, in-situ attenuated total reflectance (ATR)-FTIR measurements were performed to study the possible intermediates in CO_2_ reduction (Fig. 5a). The strong peaks at 2359 and 2313 cm^−1^ were assigned to the asymmetric stretching of CO_2_^16,58^. This is a strong hint that strong CO_2_ binding on the surface of Co-2,3-DHTA-COF was the initial step of the catalytic process^58^. The peaks at 1402 and 1455 cm^−1^ were assigned to the symmetric stretching of carbonate CO_3_^2− ^^59–62^. Interestingly, with increasing irradiation time, a characteristic peak of *COOH appeared at 1647 cm^−1^, which was reported to be the most crucial intermediate in the photoreduction process of CO_2_ to CO^48^. In addition, the gradually enhanced peak at 2130 cm^−1^ corresponded to the *CO intermediate in the photocatalytic process^63–66^, which was the critical intermediate for the formation of the final product CO. Therefore, these results suggested that *COOH and *CO intermediates were involved in the photocatalytic CO_2_RR to CO process.

To further clarify the inherent reason for the superior catalytic activity of Co-2,3-DHTA-COF and understand the nature of the active sites of the catalysts, DFT calculations were performed. For a direct comparison, the single period structures of Co-2,3-DHTA-COF (denoted as A) and Co-TP-COF (denoted as B) were selected as models to explore the reactivity (as shown in Fig. 5b). It should be noted that each A or B molecule contained two H_2_O ligands from Co(NO_3_)_2_·6H_2_O, providing two oxygen atoms to form Co-O coordination.

The calculated results indicated that **A** captured an electron first to form [**A**]^−^ and the redox potential of A (*E*_1/2_^red^ (**A**/[**A**]^−^)) was −1.39 V (vs. SHE). Then, one H_2_O ligand in [**A**]^−^ was replaced by a CO_2_ molecule, resulting in the formation of [**A**-CO_2_]^−^. The process of [**A**]^−^ + CO_2_ → [**A**-CO_2_]^−^ + H_2_O was endothermic (Δ*H* = 0.25 eV), and its Δ*G* value was 0.21 eV. From [**A**-CO_2_]^−^, one of the O sites of the CO_2_ ligand was protonated to form the intermediate **A**-COOH, i.e., *COOH species shown in situ ATR-FTIR. Then, one H^+^ + e^−^ pair (i.e., H atom) attacked the O(H) site to form unstable **A**-COOH_2_, and one H_2_O molecule was dissociated from **A**-COOH_2_ to generate **A**-CO (intermediate *CO). Finally, ligand exchange occurred between **A**-CO and an H_2_O molecule, leading to the release of a CO molecule and the regeneration of the catalyst. The rate-determining step of the CO_2_RR catalyzed by A was a ligand exchange process between [**A**]^−^ and CO_2_, and its Δ*G* was 0.21 eV. In addition, we theoretically evaluated the catalytic activity of Co-TP-COF for the CO_2_RR to CO and the computation model is presented in Fig. 5b. The rate-determining step for CO_2_RR catalyzed by **B** was the ligand exchange process between [**B**]^−^ and CO_2_ for the formation of intermediate [**B**-CO_2_]^−^. The Δ*G* value of [**B**]^−^ + CO_2_ → [**B**-CO_2_]^−^ + H_2_O was 0.50 eV, which was significantly larger than that for **A**. Therefore, the superior catalytic activity of Co-2,3-DHTA-COF occurred due to the lower energy barrier in the ligand exchange process between Co-2,3-DHTA-COF and CO_2_.

From the above experimental and theoretical results, a possible mechanism was proposed for the photoreduction of CO_2_ to CO catalyzed by Co-2,3-DHTA-COF. Upon visible light irradiation, the photosensitizer [Ru(bpy)_3_]Cl_2_ was excited, and the induced electrons were transferred to the Co-COF catalyst matrix in the Co-O_4_ coordination mode. The remaining holes were captured by the electron donor TEOA, which was then oxidized to TEOA_ox_^+^, and the entire cycle was closed. The coordination mode in Co-2,3-DHTA-COF accelerated the separation of electrons and holes, and effectively boosted the photocatalytic CO_2_ reduction to CO.

## Discussion

In summary, we designed Co-O_4_ active sites in Co-COF by modulating the position of hydroxyl groups in aldehyde modules under the guidance of a programmable approach. By using the as-designed Co-2,3-DHTA-COF as the catalyst, remarkable photocatalytic CO_2_ reduction to CO was achieved with a high CO production rate of 18,000 µmol g^−1^ h^−1^ and excellent selectivity of 95.7%. Notably, a simulation apparatus based on the optimal catalyst showed a CO production rate of 11,000 µmol g^−1^ h^−1^ and a selectivity of 89% under natural sunlight, highlighting the significant importance of Co-O_4_ sites in Co-COF for photocatalytic CO_2_RR to CO. Furthermore, it was found that a distinctive coordination environment improved the loading capability of Co (II) in the COF, accelerated the electron transfer process from photosensitizer to catalyst, and enhanced CO_2_ adsorption capacity, which boosted the catalytic performance. A detailed DFT simulation further revealed that Co-2,3-DHTA-COF enriched with Co-O_4_ active sites significantly decreased the energy barrier of the rate-determining step (ligand exchange between Co-2,3-DHTA-COF and CO_2_). Therefore, we believed that such a module programmable approach provided a promising way towards optimizing the metal coordination environment of metal-COF for highly efficient CO_2_RR and could be applied to design other photocatalysts from reticular nanomaterials.

## Methods

### Synthesis of 2,3-DHTA-TAPT-COF

In a 10 mL Pyrex tube, TAPT (62.92 mg, 0.174 mmol), 2,3-DHTA (44.08 mg, 0.260 mmol), 1,3,5-trimethylbenzene (3.4 mL), 1,4-dioxane (0.6 mL), and 6 M aqueous acetic acid (0.4 mL) were mixed and sonicated. The tube was degassed via a freeze-pump with liquid nitrogen for three cycles and then heated at 120 °С for 72 h. The produced precipitate was gathered and washed with anhydrous tetrahydrofuran (THF) for further purification. Finally, the red‒brown material was obtained with a yield of 80 wt.% by Soxhlet extraction with THF for 24 h and dried under vacuum conditions at 80 °C overnight. Other COF matrixes were synthesized with similar procedures (see Supplementary Information).

### Synthesis of Co-2,3-DHTA-COF catalyst

2,3-DHTA-TAPT-COF (50.00 mg) and Co(NO_3_)·6H_2_O (1.00 g, 3.440 mmol) were added to an ethanol/water mixed solution (v/v = 1:1) and stirred at 95 °С for 24 h. After centrifugation, the product was washed several times with water to ensure the removal of residual metal ions and then dried under vacuum, and its yield was 90 wt.%. The Co-TP-COF catalyst was synthesized with similar methods (see Supplementary Information).

### Photocatalytic CO_2_ reduction tests

The photocatalytic CO_2_ reduction experiments were conducted in a 250 mL optical reaction vessel (PQ256, Beijing Perfect light Technology Co., Ltd) with vigorous stirring in the thermostat at 25 °C. In a typical photocatalytic experiment, 1.00 mg of catalyst was dispersed in 40 mL of acetonitrile/H_2_O (v:v = 4:1) mixed solution under ultrasound. Then, 10.00 mg of photosensitizer [Ru(bpy)_3_]Cl_2_·6H_2_O and 6 mL of sacrificial reagent TEOA were dissolved in this mixture. After homogeneous mixing, the reactor was evacuated by a vacuum pump followed by purging with high-purity CO_2_ (99.999 vol.%) three times. Afterwards, CO_2_ gas was bubbled through the reactor for 30 min to fully saturate the solution at 1 atm. The light source for the photocatalytic reaction was a 300 W Xe lamp (Microsolar 300, cut 420 nm), and the light intensity was 100 mW cm^−2^ (one sun). The production of CO and H_2_ was detected by FULI 9790II gas chromatography. After a cycle of photocatalytic reduction, the catalyst was washed with water and then centrifuged for the next run. After adding an equal amount of fresh acetonitrile, H_2_O and [Ru(bpy)_3_]Cl_2_·6H_2_O, CO_2_ was reintroduced to the photocatalytic system under the same operating conditions. In addition, possible liquid products were detected by ^1^H NMR spectroscopy (Bruker AVIII HD 600 instrument at 600 MHz). Isotope labeling measurements were performed by using ^13^CO_2_ gas (enrichment, 99 at.%, Sigma‒Aldrich Co., Ltd) as the carbon source, and the gas product was identified by gas chromatography‒mass spectrometry (GC‒MS, Agilent GC/MS-7000D).

### Photoelectrochemical measurements

All photoelectrochemical measurements were conducted by a CHI 760E electrochemical workstation via a standard three-electrode cell. A saturated Ag/AgCl electrode was used as the reference electrode, and a Pt mesh was used as the counter electrode. To prepare the working electrode, appropriate amounts of catalyst sample and Nafion in ethanol were mixed and sonicated, and the mixture was spread evenly on the surface of indium tin oxide (ITO) conductive glass. After air drying, the working electrode was stored in petri dishes for related tests. In this study, an aqueous sodium sulfate solution (0.500 M) was selected as the electrolyte. In the photocurrent test, a 300 W Xe lamp (cut 420 nm) was used as a light source. For EIS measurements, the samples were tested with a frequency range from 10 kHz to 0.1 Hz. Mott-Schottky experimental plots were determined at frequencies of 500, 1000, and 1500 Hz.

### In situ ATR-FTIR

The ATR measurement was performed by a Thermo Nicolet iS50 spectrometer (USA) with a mercury cadmium telluride (MCT) detector in the system. In a typical procedure, the samples were dispersed in acetonitrile/H_2_O (v:v = 4:1) with [Ru(bpy)_3_]Cl_2_·6H_2_O and TEOA before sealing in the chamber for purging with N_2_ for 1 h. Typical signals of two intermediates were captured after the introduction of flowing CO_2_ under dark (10 min) and light irradiation (0–60 min) conditions.

### X-ray absorption fine structure (XAFS) spectroscopy

XAFS spectra (Co K-edge) were collected at the 1W1B station in the Beijing Synchrotron Radiation Facility (BSRF). The storage rings of BSRF were operated at 4–23 KeV. By using a Si(111) double-crystal monochromator, the data were collected in transmission mode using an ionization chamber (i.e., Co foil, CoO, Co_3_O_4_, and CoPc). The energy resolution of the spectrometer was approximately 1.4 eV, providing an overall energy resolution of approximately 1.5 eV at the Co K-edge, including core-hole effects. To obtain the quantitative structural parameters around central atoms, data processing was conducted by using Athena and Artemis software from the IFEFFIT package.

## Supplementary information

Supplementary Information

## Data Availability

The data that support the findings of this study are available in the paper and Supplementary Information. Source data are provided with this paper.

## Source data

Source Data
