# Supplementary material for: Designing covalent organic frameworks with Co-O4 atomic sites for efficient CO2 photoreduction
Source: Nat Commun. 2023 Feb 28;14:1147. doi: 10.1038/s41467-023-36779-4 (PMC9975230; doi:10.1038/s41467-023-36779-4)
Supplement: Supplementary file 1 — Supplementary Information [file 41467_2023_36779_MOESM1_ESM.pdf]

Supplementary Information

Designing covalent organic frameworks with Co-O_4 atomic sites for efficient CO_2 photoreduction

Qian Zhang[1,+], Shuaiqi Gao[1,+], Yingying Guo[1], Huiyong Wang[1,*], Jishi Wei[2], Xiaofang Su[1], Hucheng Zhang[1], Zhimin Liu[3,*], and Jianji Wang[1,*]

[1]Key Laboratory of Green Chemical Media and Reactions (Ministry of Education), Collaborative Innovation Center of Henan Province for Green Manufacturing of Fine Chemicals, School of Chemistry and Chemical Engineering, Henan Normal University, Xinxiang, Henan 453007, P. R. China.

[2]Department of Electrical and Computer Engineering, National University of Singapore, 4 Engineering Drive 3, 117583, Singapore.

[3]Beijing National Laboratory for Molecular Sciences, Key Laboratory of Colloid, Interface and Thermodynamics, CAS Research/Education Centre for Excellence in Molecular Sciences, Institute of Chemistry, Chinese Academy of Sciences, Beijing 100190, China.

[+]These authors contributed equally.

*Correspondence authors.
Email: hywang@htu.edu.cn (H.Y. W.); liuzm@iccas.ac.cn (Z.M. L.); jwang@htu.edu.cn (J.J. W.)

Table of Contents:

1. Supplementary Methods……………………………S2

2. Supplementary Figures 1-36…………………………..S7

3. Supplementary Tables 1-8……………………………S42

4. Supplementary References……………………………S46

1. Supplementary Methods

Chemicals and COF synthesis

Chemicals

$Co(NO_3)_2·6H_2O$ (98 %, Alfa Aesar), 2,4,6-tris(4-aminophenyl)-1,3,5-triazine (TAPT, 98%, Yanshen Technology), 2,4,6-trihydroxybenzene-1,3,5-tricarbaldehyde (TP, 97%, Yanshen Technology), 2,3-dihydroxybenzene-1,4-dicarboxaldehyde (2,3-DHTA, 97%, Alfa Aesar), acetonitrile (MeCN, 99%, Keshi), ethanol (99.7%, Keshi), triethanolamine (TEOA, 99%, Aladdin), tris(2,2'-bipyridyl) ruthenium(Ⅱ) chloride hexahydrate (98%, Macklin), 1,3,5-trimethylbenzene (98%, Aladdin), methanol (99%, Keshi), 1,4-dioxane (99.7%, Damas-beta), tetrahydrofuran (THF, 99%, Deen reagent), Ar gas (99.99 vol%, Xinxiang Yuxin Gas Manufacturing Co., Ltd) and CO_2 gas (99.999 vol%, Xinxiang Yuxin Gas Manufacturing Co., Ltd) were used in the present work.

Synthesis of TP-COF

In a 10 mL Pyrex tube, TAPT (70.60 mg, 0.200 mmol), TP (42.00 mg, 0.200 mmol), 1,3,5-trimethylbenzene (1.5 mL), 1,4-dioxane (1.5 mL) and 6 M aqueous acetic acid (0.25 mL) were mixed and sonicated until uniform dispersion. The tube was degassed via freeze-pump with liquid nitrogen for three cycles and heated at 120 °C for 96 h. The precipitate was collected through a 50 mL centrifuge tube and washed with anhydrous THF and ethanol for further purification. Finally, the red powder was obtained by Soxhlet extraction with THF for 24 h and dried under vacuum at 80 °C overnight with a yield of 70 wt.%.

Materials characterization

Fourier transform infrared (FTIR) spectra were recorded on a Perking Elmer Spectrum 400F spectroscopy. X-ray diffraction (XRD) patterns of all sample powders were measured by a X'Pert3 Powder Advance diffractometer with monochromatized Cu Kα radiation operating at 45 kV and 40 mA. Field emission scanning electron microscope (FESEM) images were obtained on JEOL JSM-6390L. Spherical aberration corrected transmission electron microscope (TEM) at an accelerating voltage of 80 kV(JEM-

ARM300F). Nitrogen adsorption and desorption isotherms were measured at 77 K by Quantachrome Autosorb-iQ adsorption analyzer after degassing at 200 ^{o}C for 8 h. Specific surface areas and pore size distributions were determined by Brunauer-Emmet-Teller (BET) model and nonlocal density functional theory (NLDFT) model, respectively. CO_2 adsorption isotherms were measured at 298 K by using a Quantachrome Autosorb-iQ adsorption analyzer after degassing at 200 ^{o}C for 12 h. CO_2 temperature-programmed desorption (CO_2-TPD) was performed on an Autochem 2920 II chemisorption analyzer (Micromeritics) equipped with a thermal conductivity detector. The samples (100 mg) were pretreated under He atmosphere at 150 ^{o}C for 1 h (temperature increasing rate for 10 ^{o}C min^{-1}), and then cooled to 50 ^{o}C. After this procedure, CO_2 was absorbed by COF for 30 min at 50 ^{o}C, and then CO_2 desorption was analyzed at different temperature (from 50 to 350 ^{o}C). The CO_2-TPD curve was obtained by a TCD detector. X-ray photoelectron spectroscopy (XPS) measurements were performed on the Thermo Scientific K-Alpha electron energy spectrometer with Al Kα (1486.6 eV) radiation as the X-ray excitation source. All binding energies were referenced to the C 1s peak (284.6 eV) based on adventitious carbon.

The inductively coupled plasma-mass spectrum (ICP-MS) of metal elements was recorded on the Agilent 7700 spectroscopy. Electrochemical tests were performed by the CHI 760E electrochemical working station (Shanghai). The steady-state photoluminescence (PL) spectra and PL decay spectra were measured by FLS980 Fluorescence Spectrometer (UK). Solid-state UV-vis diffuse reflectance spectra of the samples were collected on a Perkin Elmer Lambd 950 spectroscopy (USA) using $BaSO_4$ as the reference standard. Thermogravimetric analysis (TGA) was conducted from 25 ^{o}C to 800 ^{o}C under N_2 protection with a heating rate of 10 ^{o}C min^{-1} using a NETZSCH STA449C thermal analyzer. The ultra-fast femtosecond time-resolved transient absorption (fs-TA) spectra were measured by an Helios pump-probe system (Ultrafast Systems LLC) coupled with an amplified femtosecond laser system (Coherent, 35 fs, 1kHz, 800 nm). The probe pulses (from 400 to 700 nm) were produced by focusing a small portion (around 10 μJ) of the fundamental 800 nm laser pulses into 1 mm thickness of rotated

CaF_2. The 365 nm pump pulses were generated from an optical parametric amplifier (TOPAS-800-fs).

The effect of different dosages of Co-2,3-DHTA-COF on the CO production

The photocatalytic CO_2RR experiments with different dosages of Co-2,3-DHTA-COF (0.50, 1.00, 1.50, 2.00, 5.00 and 10.00 mg) were completed to systematically examine the effect of catalyst dosage on the production of CO. It was found that the CO production in 4 hours was 0.078, 0.072 and 0.072 mmol when the amount of Co-2,3-DHTA-COF used in this photocatalytic system was 0.50, 1.00 and 1.50 mg, respectively, indicating that the increase in catalyst dosage did not significantly affect CO production. However, the CO production decreased to 0.060, 0.054 and 0.051 mmol when the dosage of Co-2,3-DHTA-CO was increased to 2.00, 5.00 and 10.00 mg. This result suggests that when catalyst dosage was >1.50 mg, the catalytic performance of the COF powder was reduced, which means that the catalyst did not fully take effect in the catalysis.

In fact, the CO_2 photoreduction by COF is a heterogeneous reaction and was conducted at the gas-solid-liquid interfaces. Importantly, the COF material is very light and fluffy, which usually floats on the top of reaction mixture in the liquid-solid mode reaction. Even under stirring, the contact of COF catalyst with components in the catalytic system is very limited. These results in two main problems: one is the low light utilization efficiency due to the strong light-shading effect; and another problem is the limited contact of the COF catalyst with the reactant and intermediate. Thus, the observed decrease in catalytic efficiency is not only related to the light-shading effect due to the increased dosage of the COF powder, but also related to the limited contact area of the catalyst with the reactant and intermediate[1]. In other words, optimal dosage of COF catalyst is necessary for the photoreduction reaction, however, excess catalyst would have a negative effect for the reaction owing to the factors mentioned above. This may be the possible reason why low dosage of catalysts was usually used in the previous studies and in this work

Error analysis of CO production rate

In order to obtain reliable CO production rate data, accurate mass determination of photocatalyst Co-2,3-DHTA-COF is very important. Therefore, a high precision

electronic balance with resolution of ±0.00001g (±0.01mg) was used in this work. We estimated the related experimental errors in weighing and production rate of CO by performing five independent reduction experiments catalyzed by Co-2,3-DHTA-COF, and the result was listed in Supplementary Table 8. The calculated standard deviation was ± 0.02 mg for the mass of Co-2,3-DHTA-COF and ± 0.210 $mmol\cdot g^{-1}\cdot h^{-1}$ for the CO production rate.

Turnover number and CO selectivity

The turnover number (TON) was calculated by supplementary Eq. S1[2-3]:

$$TOF = n_{co}/(n_{active\ site}\ t) \quad (S1)$$

where n_{co} and $n_{active\ site}$ are molar number of the CO product and active sites of the photocatalyst, respectively, and t is the reaction time (hour). The selectivity of CO was calculated by Eq. S2:[4]

$$Selectivity(CO)\% = n_{co}\times 100\%/(n_{co}+n_{H_2}) \quad (S2)$$

where n_{co} and n_{H_2} refer to the molar number of CO and H_2 products under visible light irradiation (λ > 420 nm).

Apparent quantum efficiency

The apparent quantum efficiency (AQE) of the catalysts was measured by different bandpass filters (including 420 nm, 450 nm, 500 nm, 520 nm, 550 nm, 600 nm and 630 nm) under the same photocatalytic reaction conditions, and the light intensity was detected by a Newport 91150-2000 optical power meter (USA Newport corporation). The AQE values were calculated by Eq. S3[5-6]:

$$AQE(\%) = (\text{Total consumed electron number}/\text{incidented photon number})\times 100\%$$

$$= (2\times n_{co}\times N_A\times h\times c)\times 100\%/(S\times P\times T\times \lambda) \quad (S3)$$

where n_{co} is the molar number of the CO, N_A is Avogadro's constant (6.022×10^{23} mol^{-1}), h is the Planck’s constant (6.63×10^{-34} m^2 kg s^{-1}), c is the speed of light (3×10^8 m s^{-1}), S is the irradiation area (cm^2), P is irradiation intensity (W cm^{-2}), t is irradiation time (s), and λ is the wavelength of the light source, respectively.

DFT calculations

All the calculations were performed at the DFT level by using the B3LYP-D3 hybrid

functional[7,8]. In B3LYP-D3, a semiempirical dispersion potential to the traditional Kohn pseudo-DFT energy was included. For metal Co, the LANL2DZ basis set was used[9], while the basis set 6-31G(d, p) was used for nonmetals H, C, N and O elements[10]. Geometry optimizations were conducted with the CPCM solvation model (CH_3OH, ε = 32.70)[11]. All the DFT calculations were carried out by the Gaussian 09 program[12]. The redox potential $E_{1/2}$ corresponding to the half-reaction (O + ne → R) was given by the Nernst equation[13]:

$$E_{1/2} = -\Delta G_{\mathrm{m}}^{\theta}/\mathrm{n}F - E_{\mathrm{SHE}}^{\theta} \qquad \text{(S4)}$$

where $\Delta G_{\mathrm{m}}^{\theta}$ is the standard Gibbs free energy change of the half-reaction (O + ne → R), F is the Faraday constant, and $E_{\mathrm{SHE}}^{\theta}$=4.28 V is potential of the referenced standard hydrogen electrode.

2. Supplementary Figures 1-36

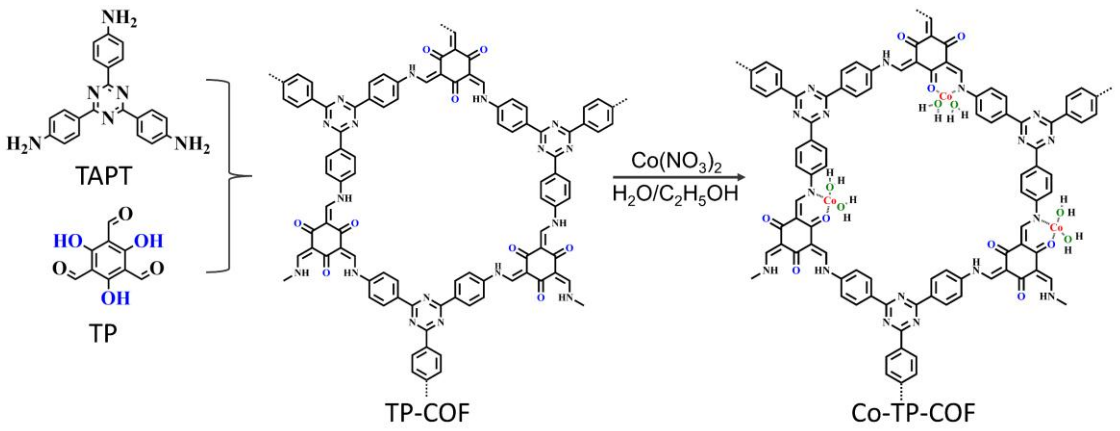

Supplementary Figure 1 Synthesis. Schematic illustration for the synthesis of TP-COF and Co-TP-COF.

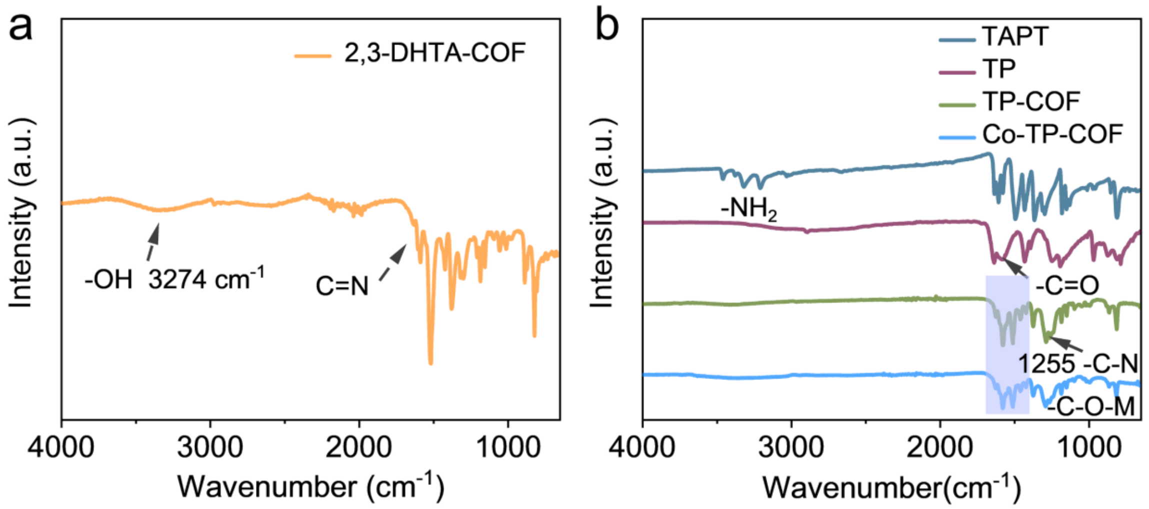

Supplementary Figure 2 FTIR spectra. **a** 2,3-DHTA-COF. **b** Co-TP-COF, TP-COF, TAPT and TP.

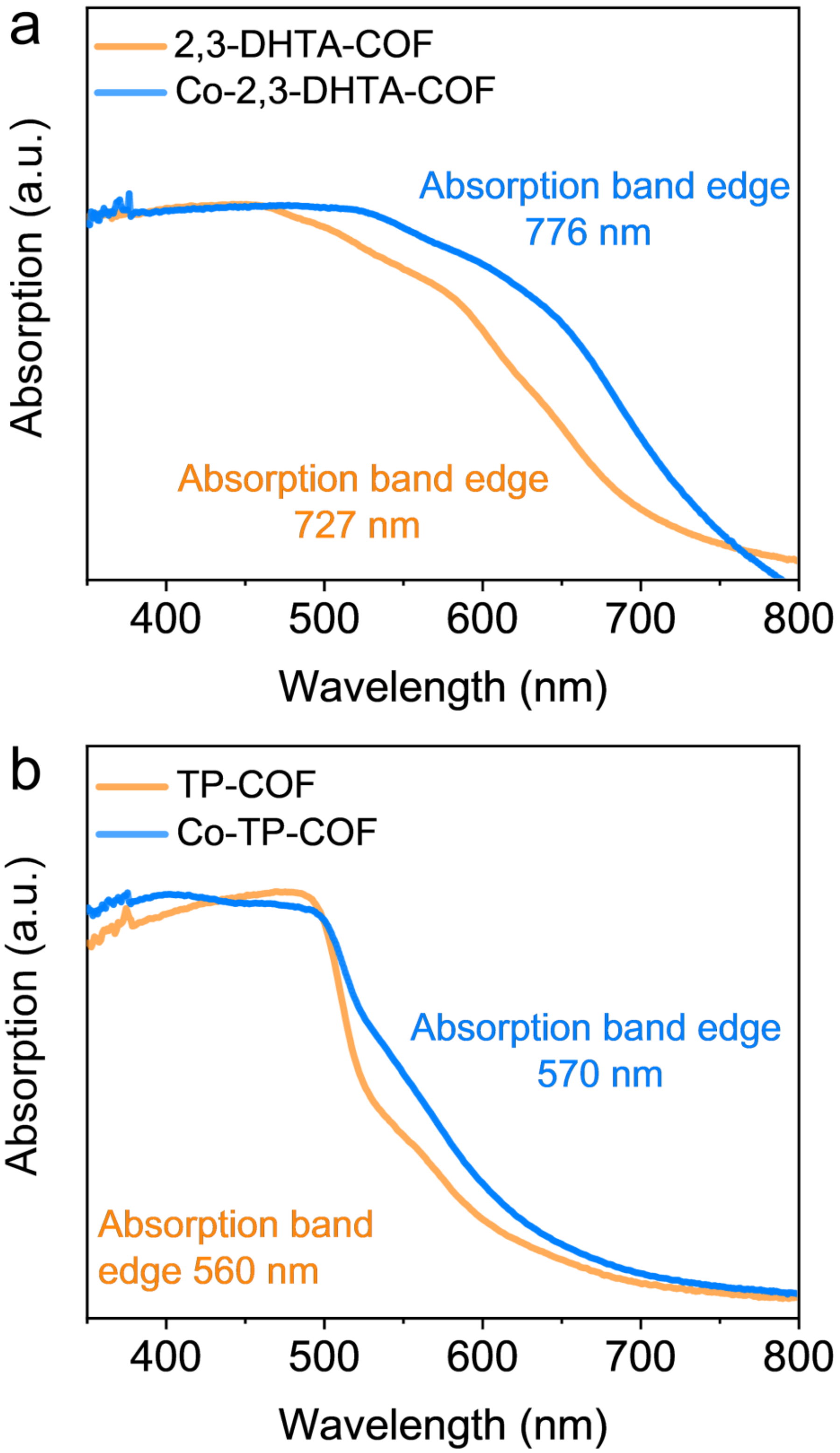

Supplementary Figure 3 Solid-state UV-vis diffuse reflectance spectra. a 2,3-DHTA-COF and Co-2,3-DHTA-COF. **b** TP-COF and Co-TP-COF.

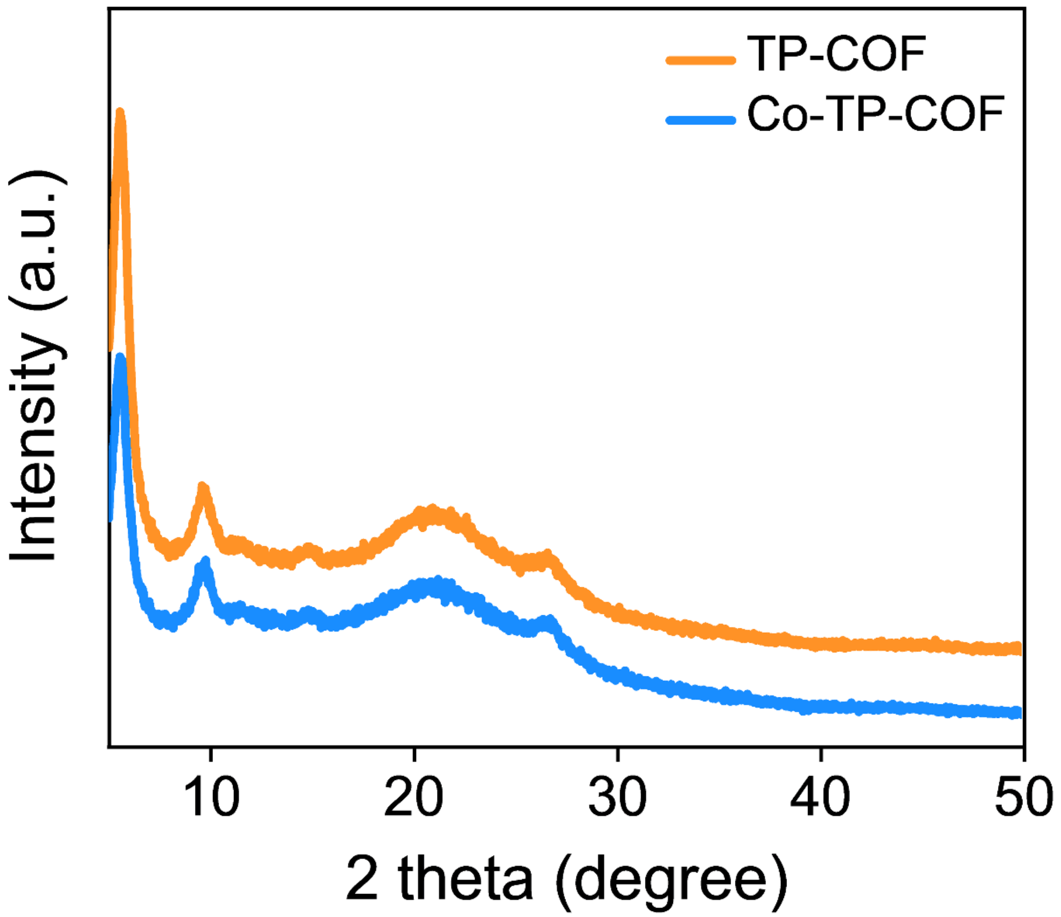

Supplementary Figure 4 PXRD. Experimental PXRD patterns of TP-COF and Co-TP-COF.

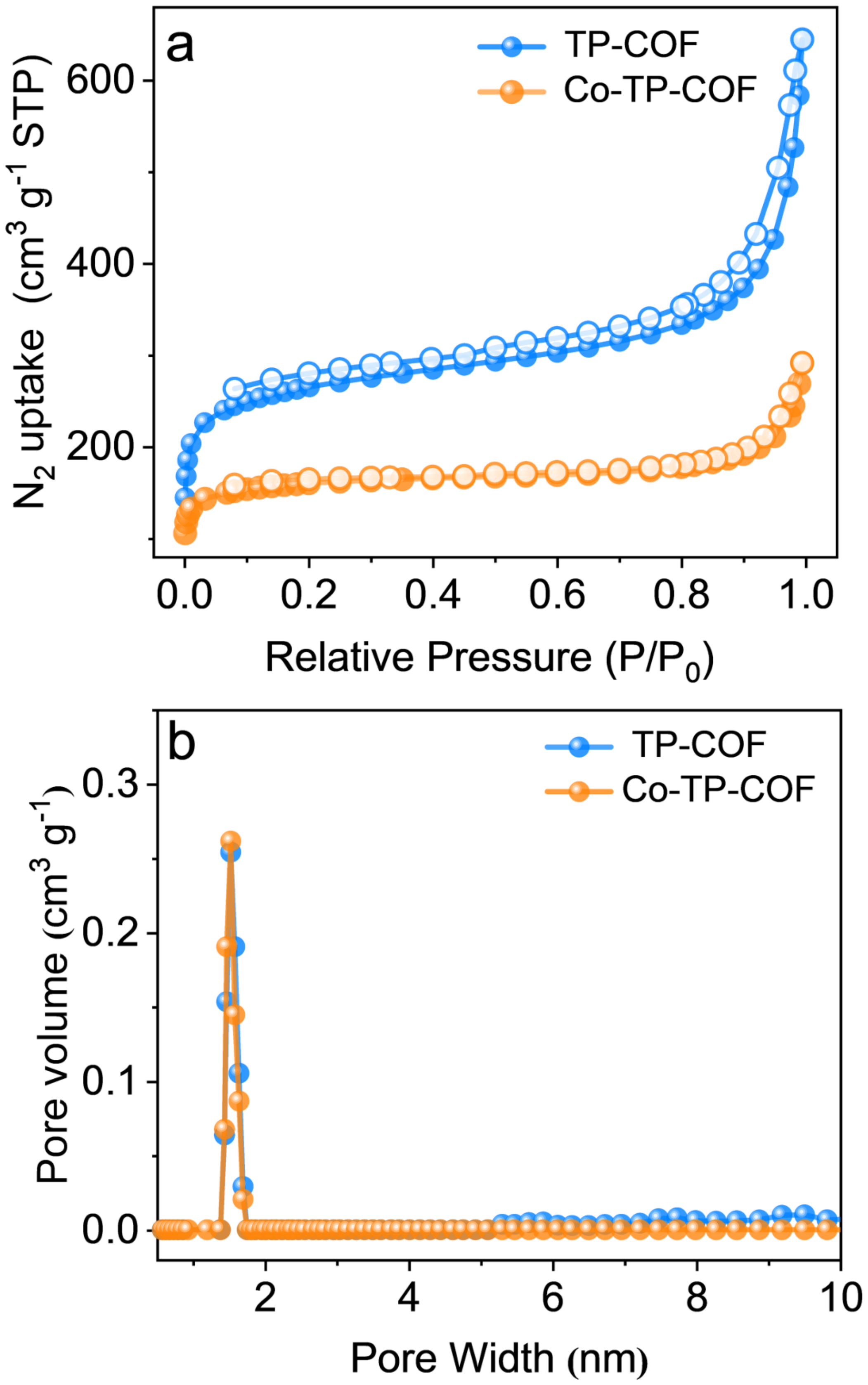

Supplementary Figure 5 Nitrogen adsorption isotherms and pore size distribution. a Nitrogen adsorption isotherms. **b** Pore size distribution of TP-COF and Co-TP-COF.

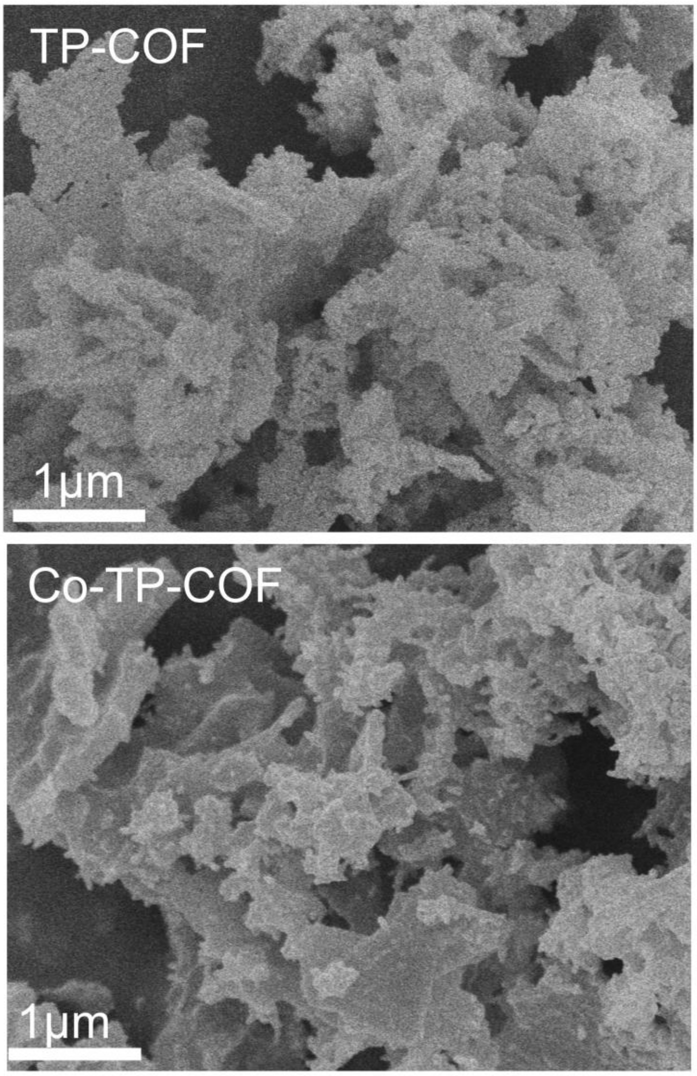

Supplementary Figure 6 SEM images. SEM images of TP-COF and Co-TP-COF.

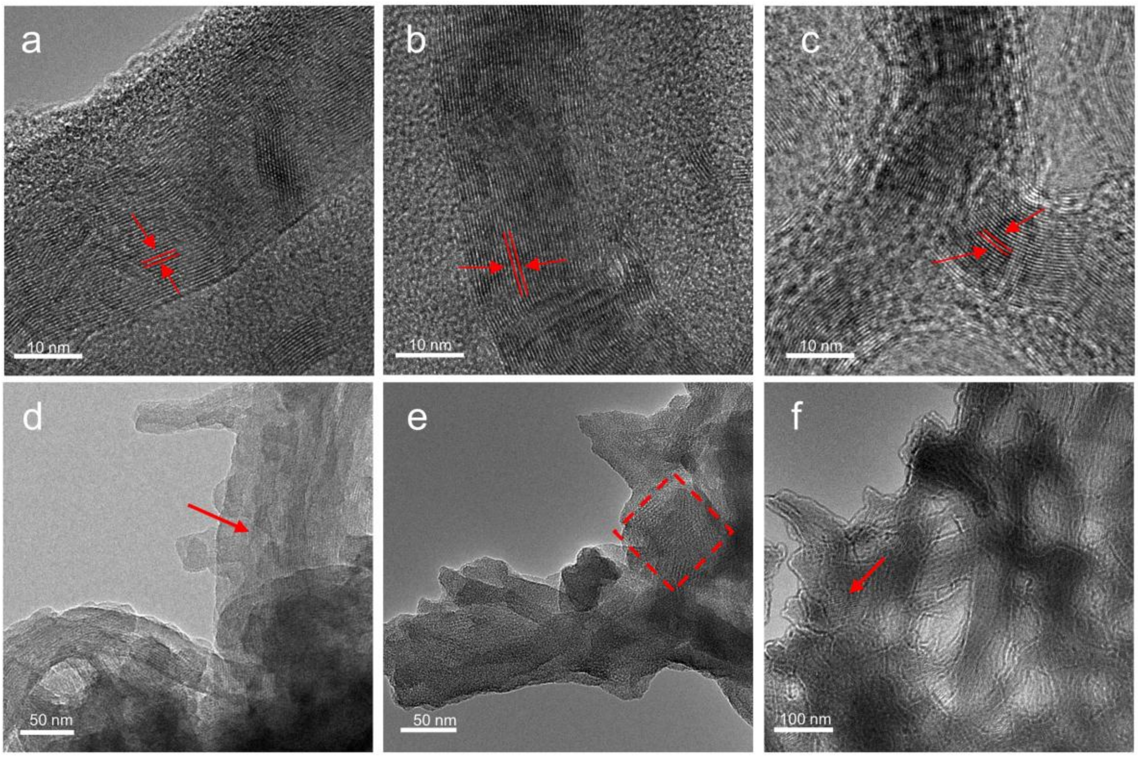

Supplementary Figure 7 High-resolution TEM images of Co-2,3-DHTA-COF. a-c The stacked layer in different locations. **d-f** The pore channel.

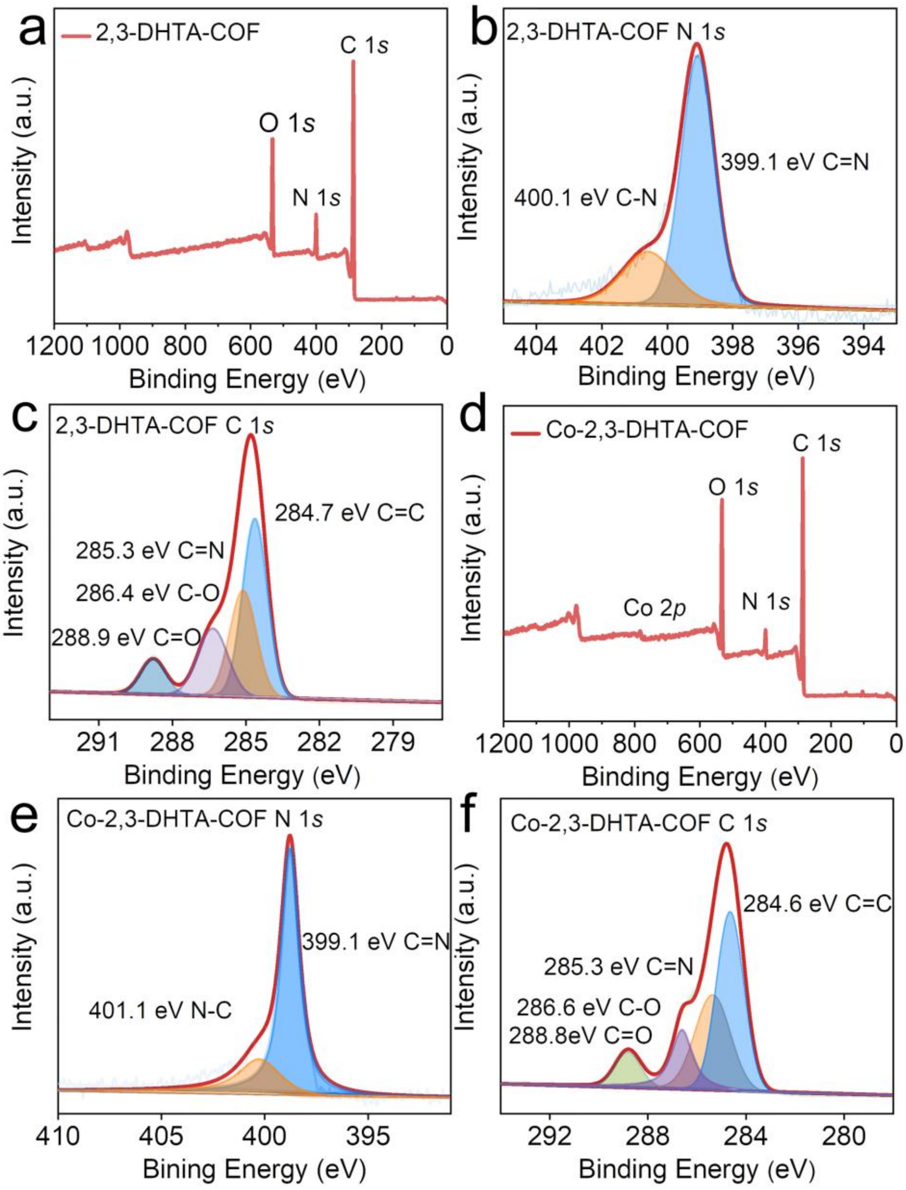

Supplementary Figure 8 XPS survey spectra of 2,3-DHTA-COF and Co-2,3-DHTA-COF. **a** XPS survey spectrum for 2,3-DHTA-COF. **b, c** High-resolution XPS spectra of N 1*s* and C 1*s* for 2,3-DHTA-COF. **d** XPS survey spectrum for Co-2,3-DHTA-COF. **e, f** High resolution XPS spectra of N 1*s* and C 1*s* for Co-2,3-DHTA-COF.

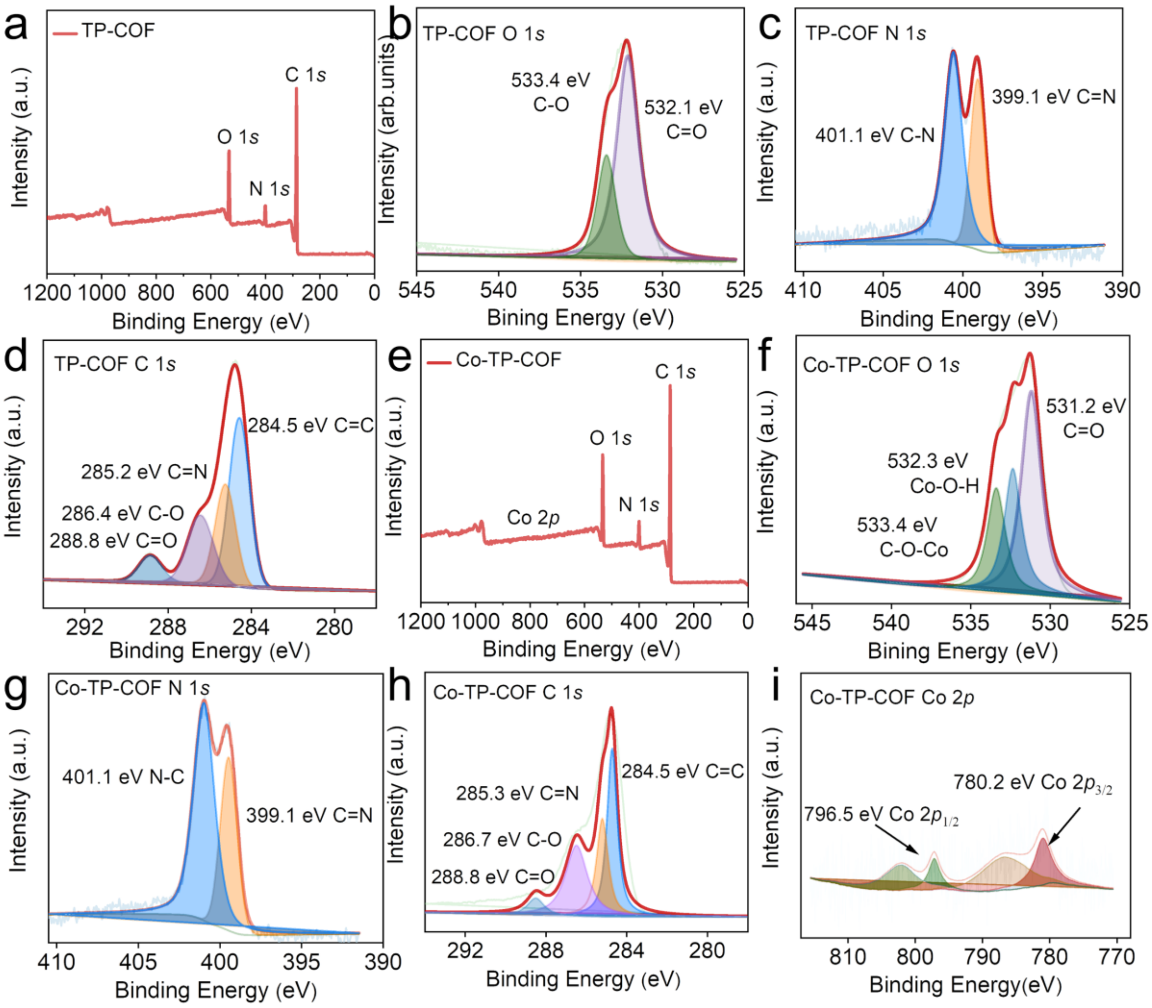

Supplementary Figure 9 XPS survey spectra of TP-COF and Co-TP-COF. a XPS survey spectrum for TP-COF. **b-d** High-resolution XPS spectra of O 1*s*, N 1*s*, and C 1*s* for TP-COF. **e** XPS survey spectrum for Co-TP-COF. **f-i** High-resolution XPS spectra of O 1*s*, N 1*s*, C 1*s* and Co 2*p* for Co-TP-COF.

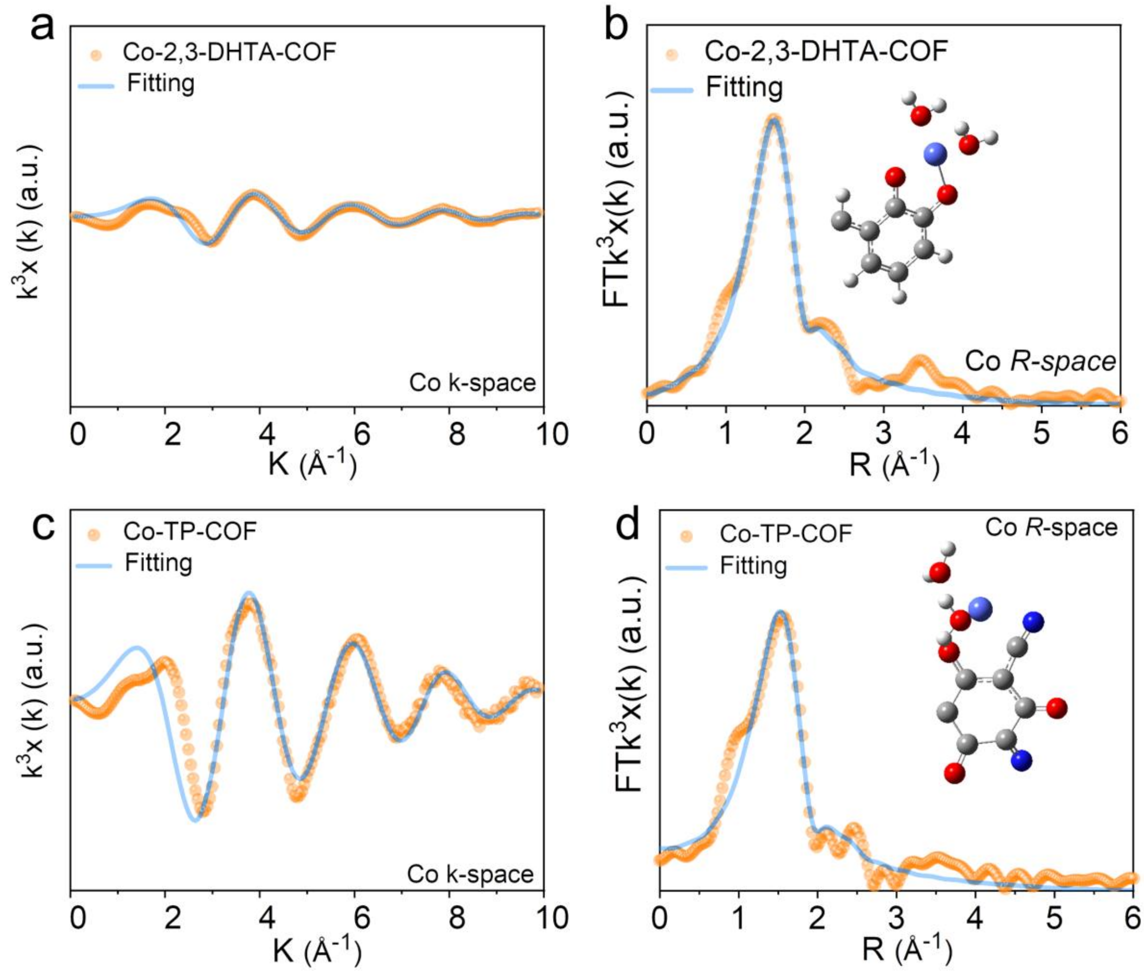

Supplementary Figure 10 EXFAS fitting curves. EXFAS fitting curves for Co-2,3-DHTA-COF: **a** In k-space. **b** In R-space; EXFAS fitting curves for Co-TP-COF. **c** In k-space. **d** In R-space.

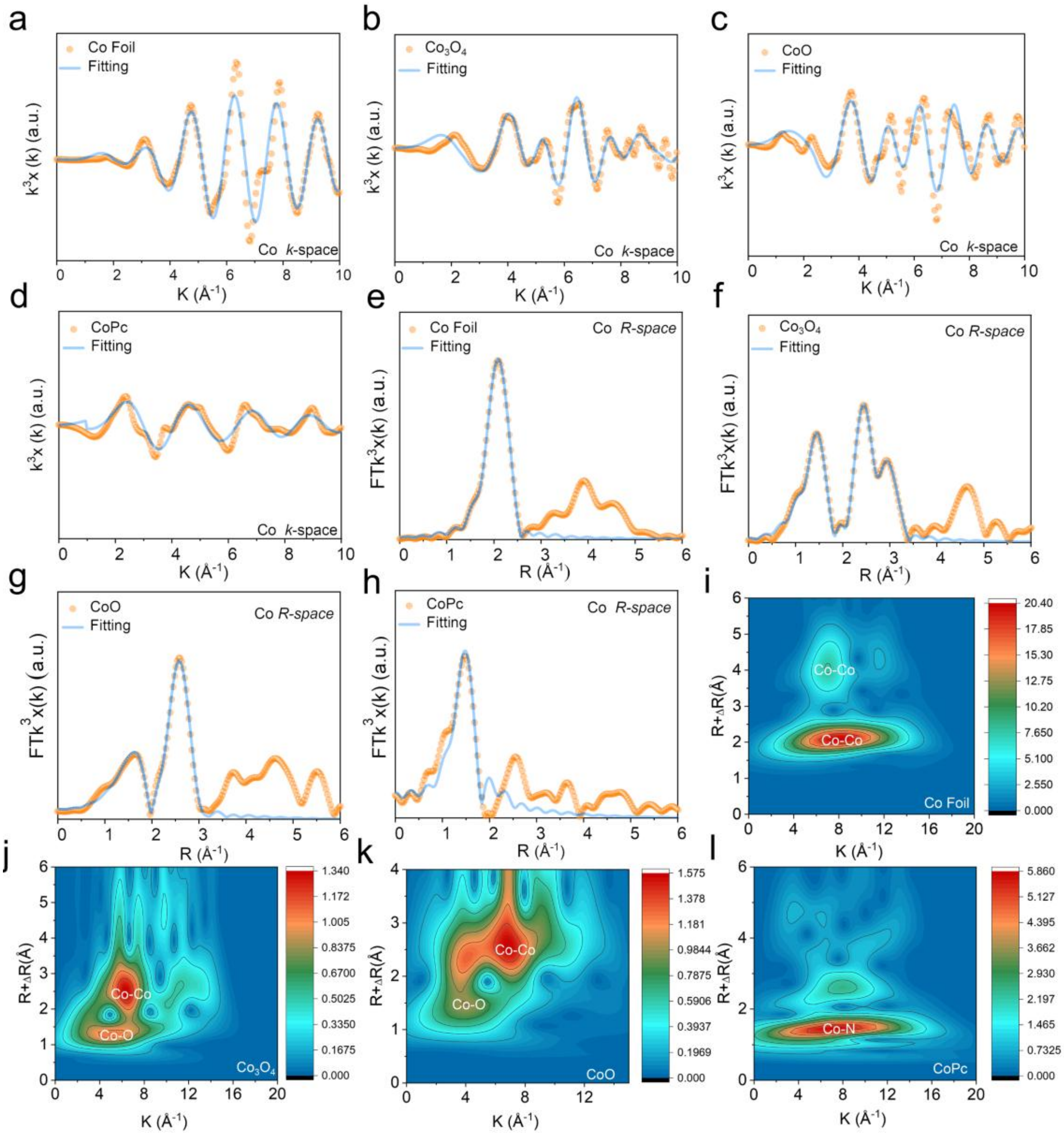

Supplementary Figure 11 EXFAS fitting curves and WT-EXAFS spectra for standard samples. EXFAS fitting curves for **a** Co foil. **b** Co_3O_4. **c** CoO and **d** CoPc in k-space. **e** Co foil. **f** Co_3O_4. **g** CoO and **h** CoPc in R-space. WT-EXAFS spectra in discriminating radial distance and k-space resolution of standard sample: **i** Co foil. **j** Co_3O_4. **k** CoO, and **l** CoPc.

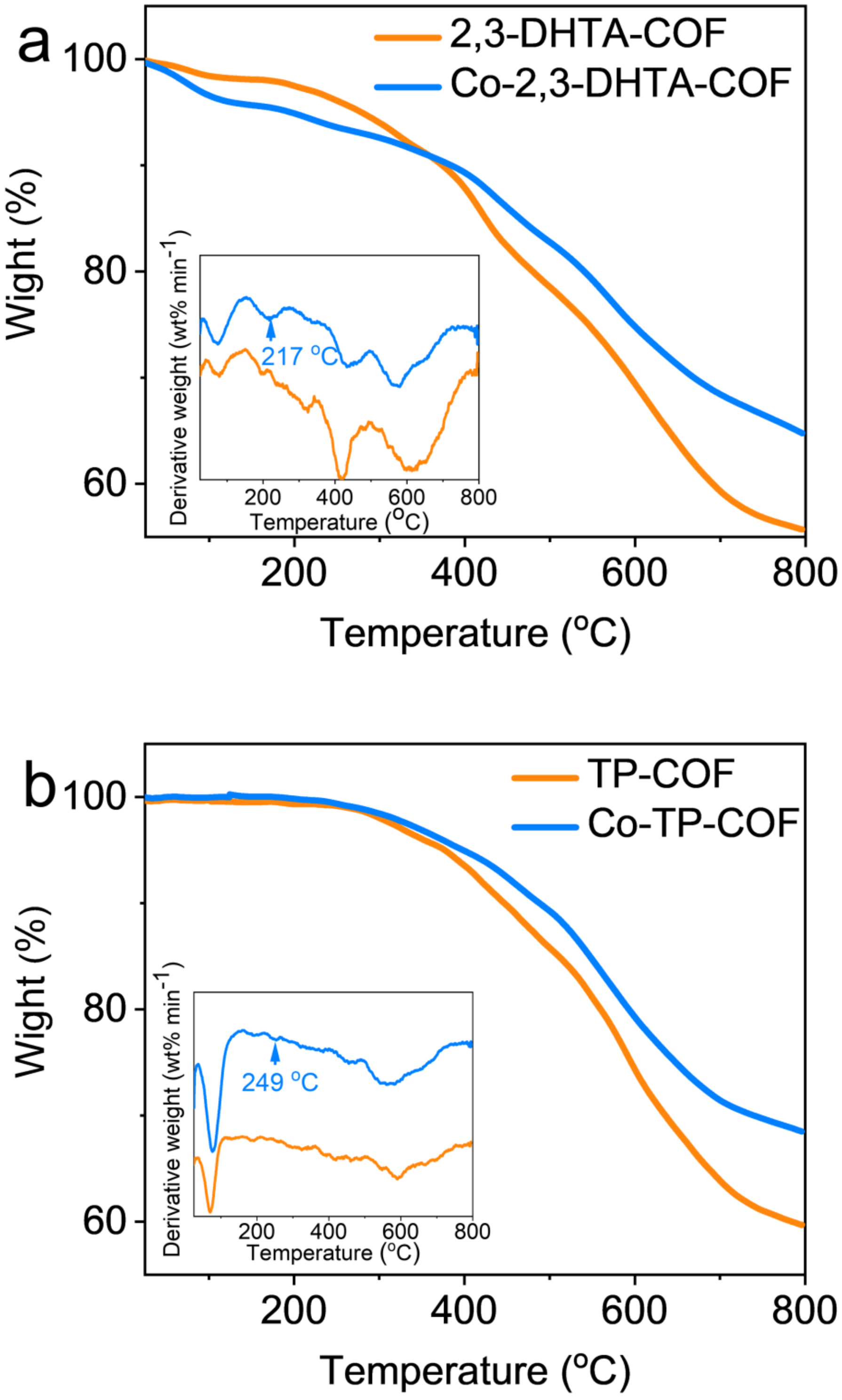

Supplementary Figure 12 TGA. TGA curves of **a** Co-2,3-DHTA-COF and 2,3-DHTA-COF, **b** Co-TP-COF and TP-COF. Inset: the thermal peaks of crystalline water in the COFs and Co-COFs calculated from the first derivative of the TGA curves.

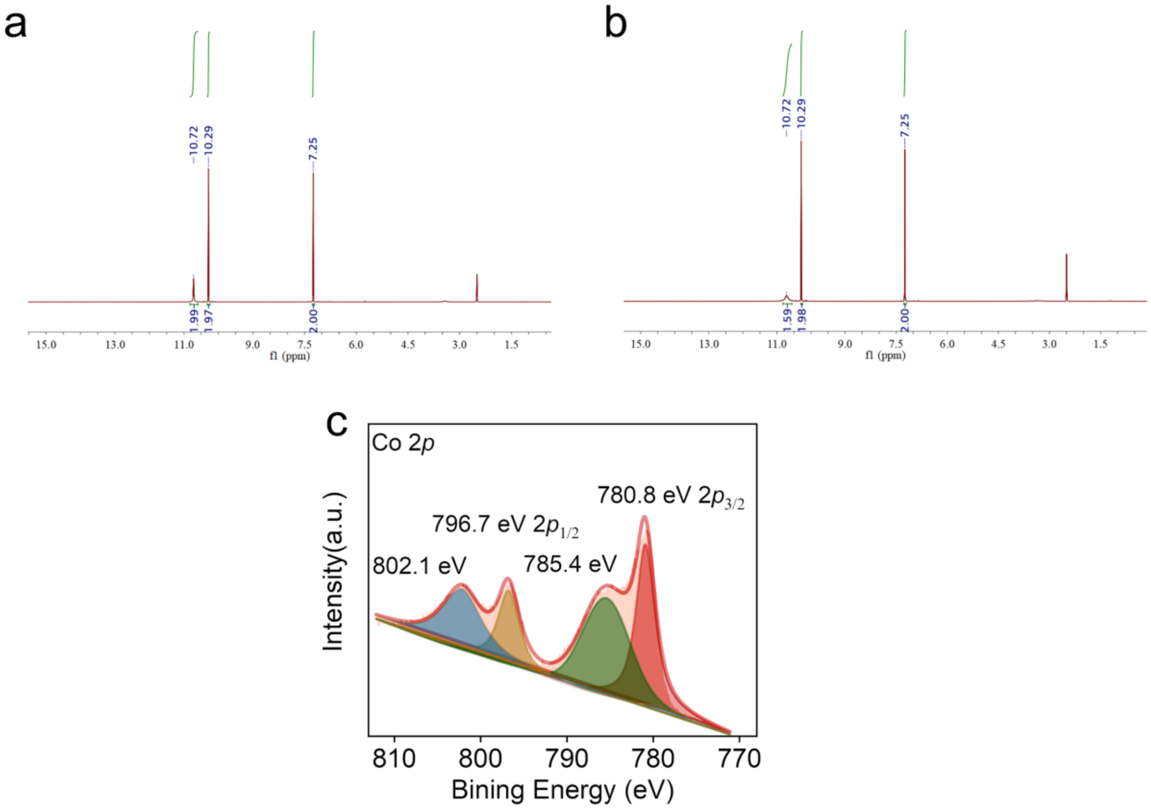

Supplementary Figure 13 ^{1}**H NMR spectra and High-resolution XPS spectra of Co 2*p*.** ^{1}H NMR spectra of **a** 2,3-dihydroxybenzene-1,4-dicarboxaldehyde (2,3-DHTA). **b** Co-2,3-dihydroxybenzene-1,4-dicarboxaldehyde (Co-2,3-DHTA) in d^{6}-DMSO. **c** High-resolution XPS spectra of Co 2*p* in Co-2,3-dihydroxybenzene-1,4-dicarboxaldehyde complex.

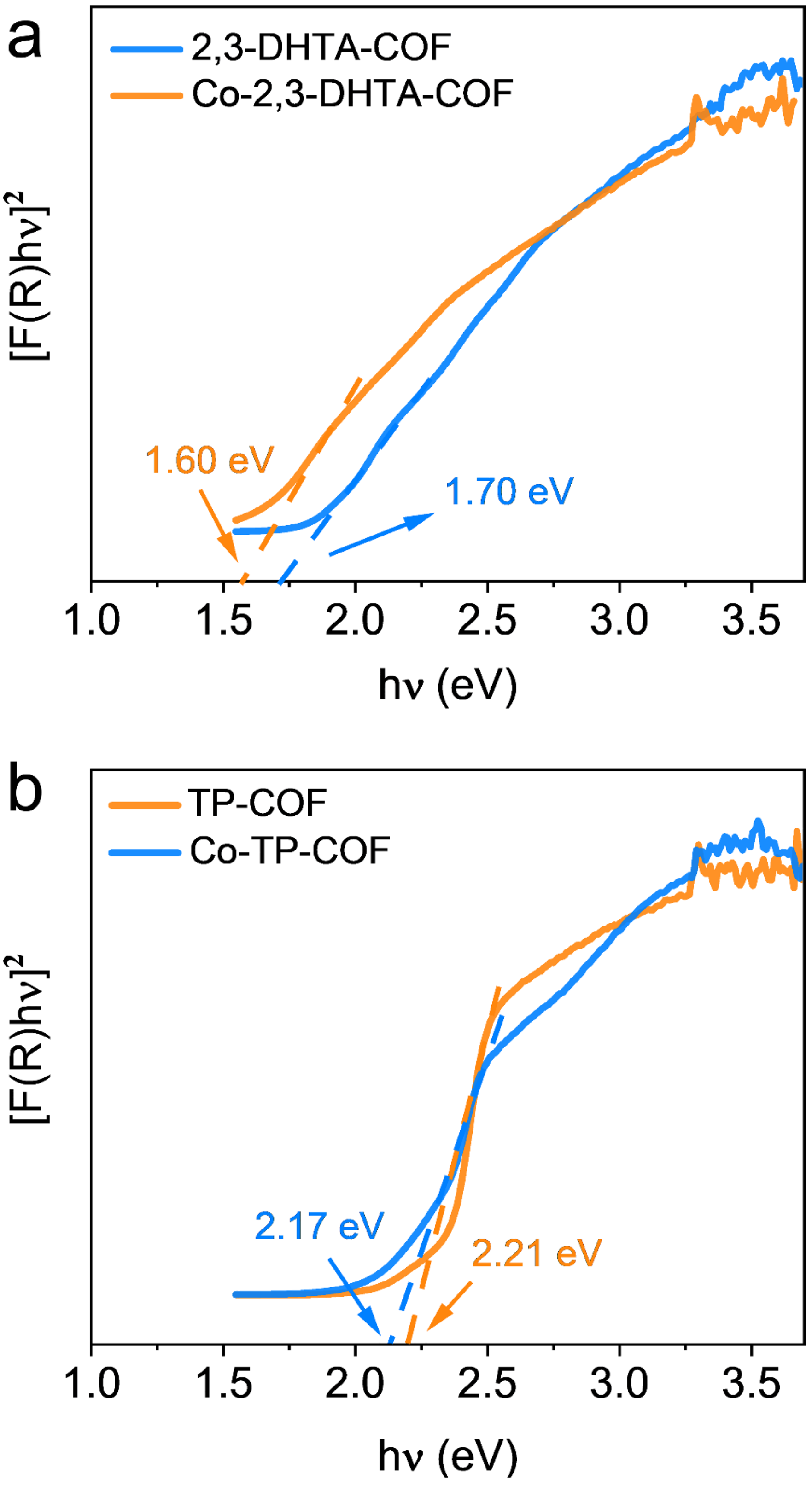

Supplementary Figure 14 Tauc plots of Co-COF/COF derived from solid-state UV-vis diffuse reflectance spectra in Supplementary Fig. 3. a 2,3-DHTA-COF and Co-2,3-DHTA-COF. **b** TP-COF and Co-TP-COF.

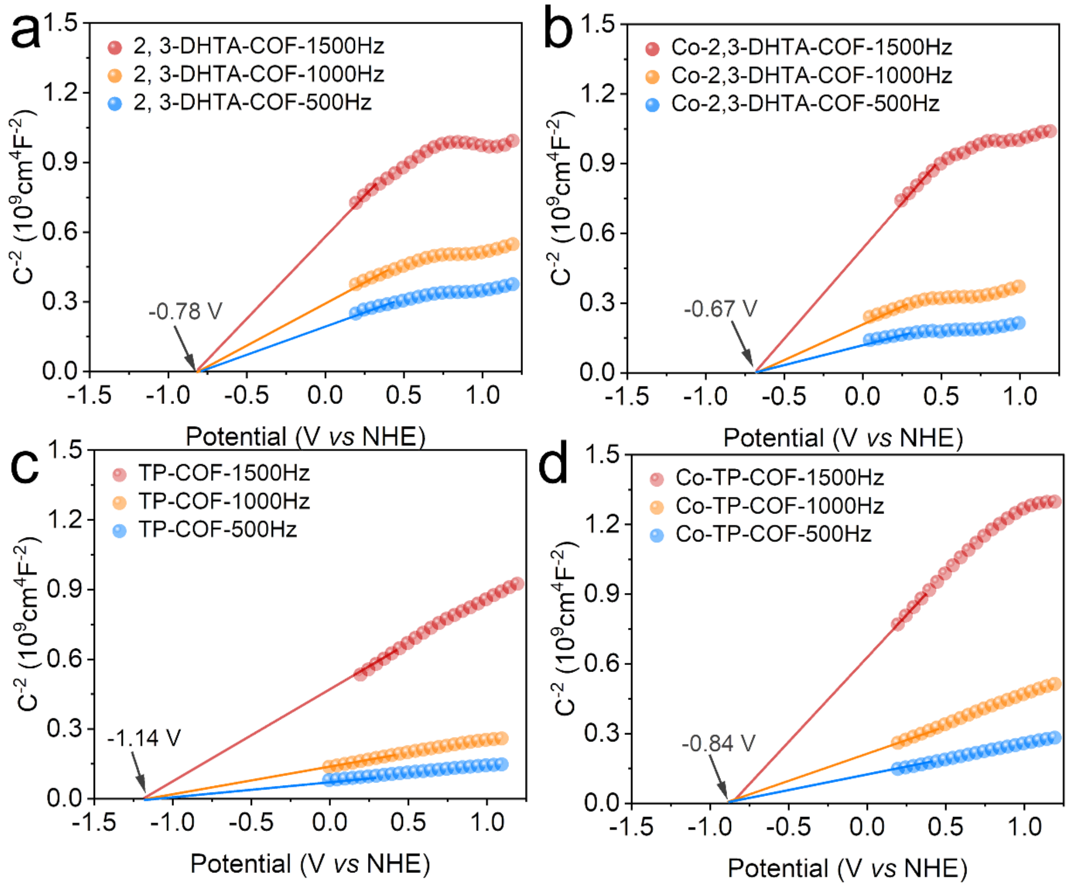

Supplementary Figure 15 Mott-Schottky plots at different frequencies (500, 1000, and 1500 Hz). a 2,3-DHTA-COF. **b** Co-2,3-DHTA-COF. **c** TP-COF. **d** Co-TP-COF.

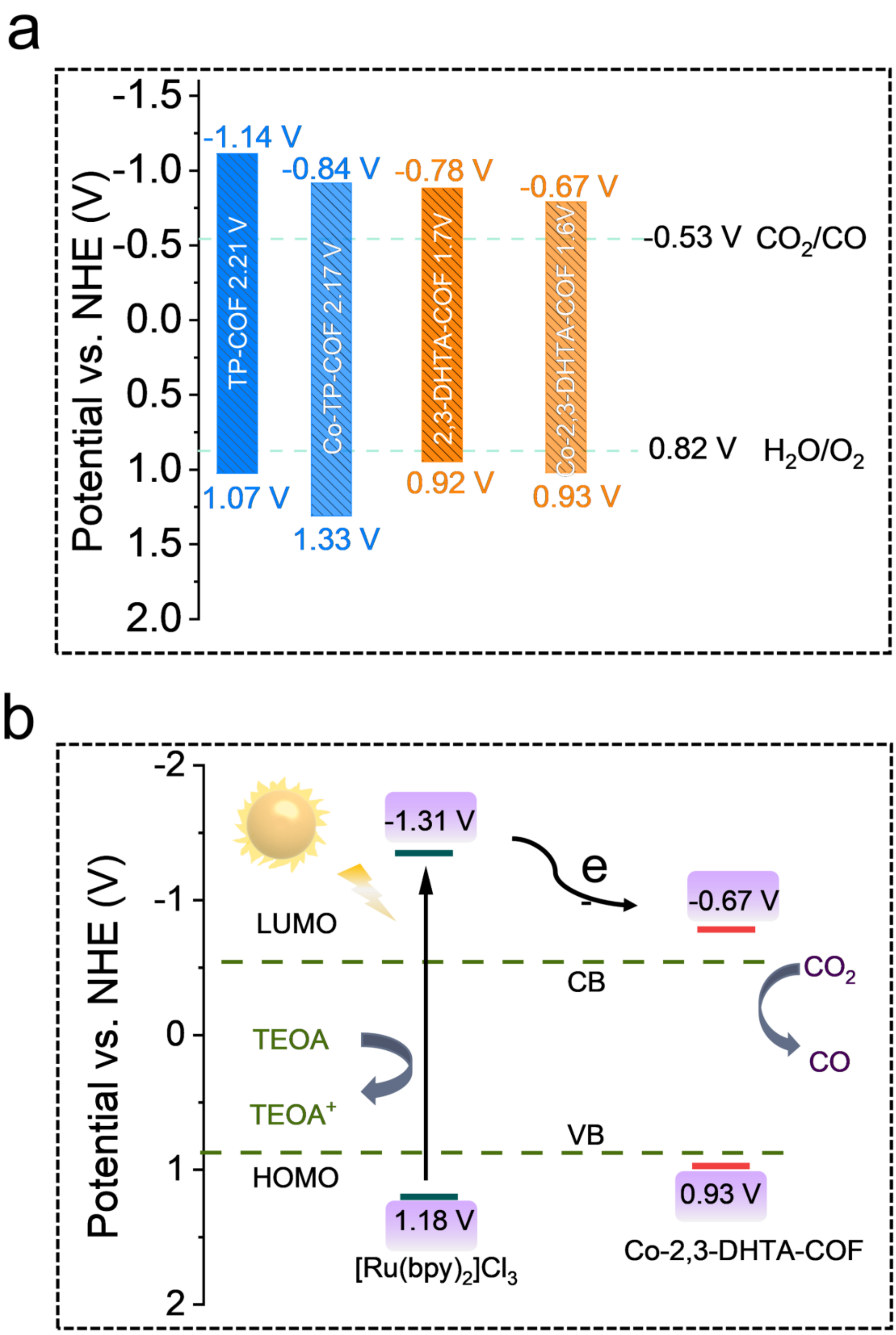

Supplementary Figure 16 Band structures and mechanism for CO_2 photoreduction. **a** Band gap structures of the COFs and Co-COFs studied in this work. **b** The proposed mechanism for CO_2 photoreduction by optimal Co-2,3-DHTA-COF.

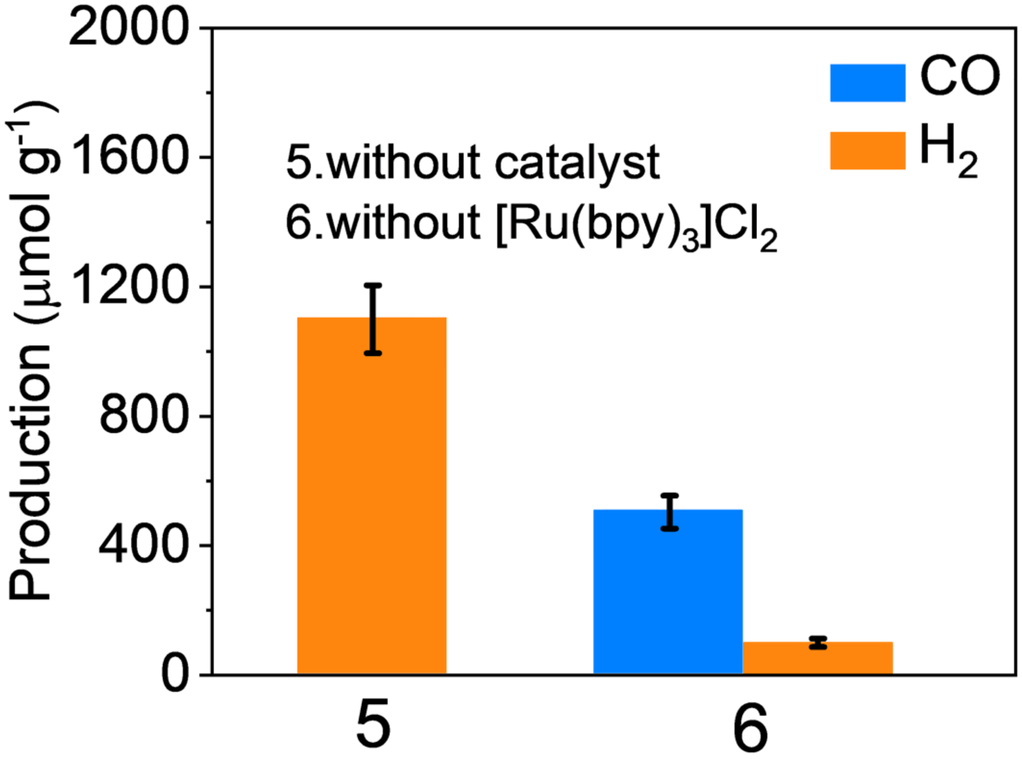

Supplementary Figure 17 Catalytic products under different conditions.

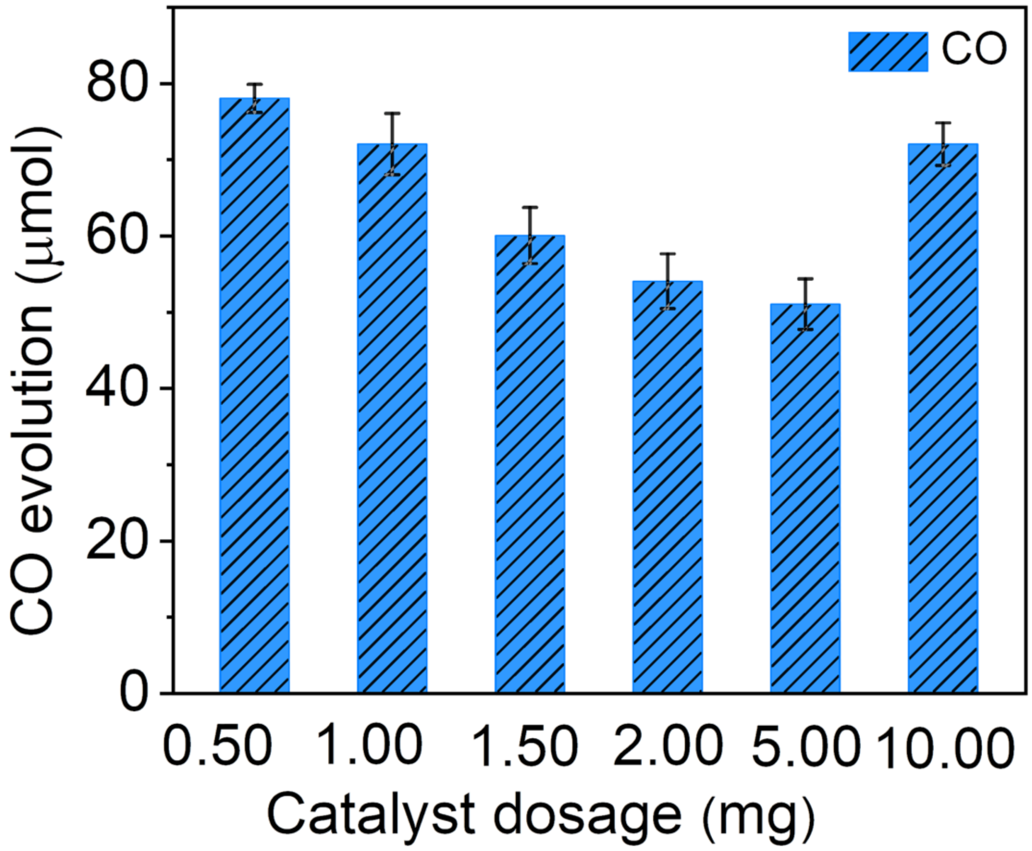

Supplementary Figure 18 Mass-loading for CO_2 photoreduction. Dependence of catalyst mass-loading on photocatalytic CO production within 4 h. The error bars for CO evolution uncertainty represent one standard deviation based on three independent samples.

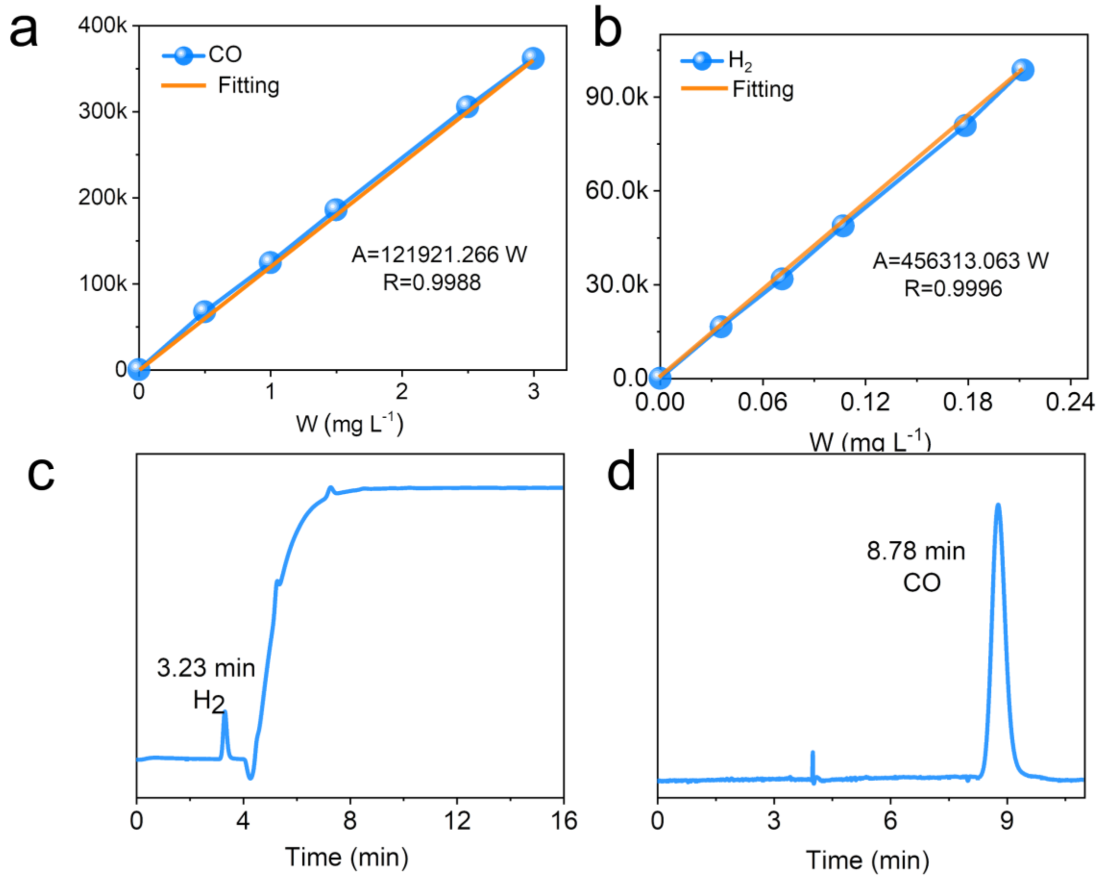

Supplementary Figure 19 GC analysis. Linear GC calibration plots of **a** CO and **b** H_2. The corresponding retention time based on the GC profile of the optimal Co-2,3-DHTA-COF: **c** H_2, **d** CO.

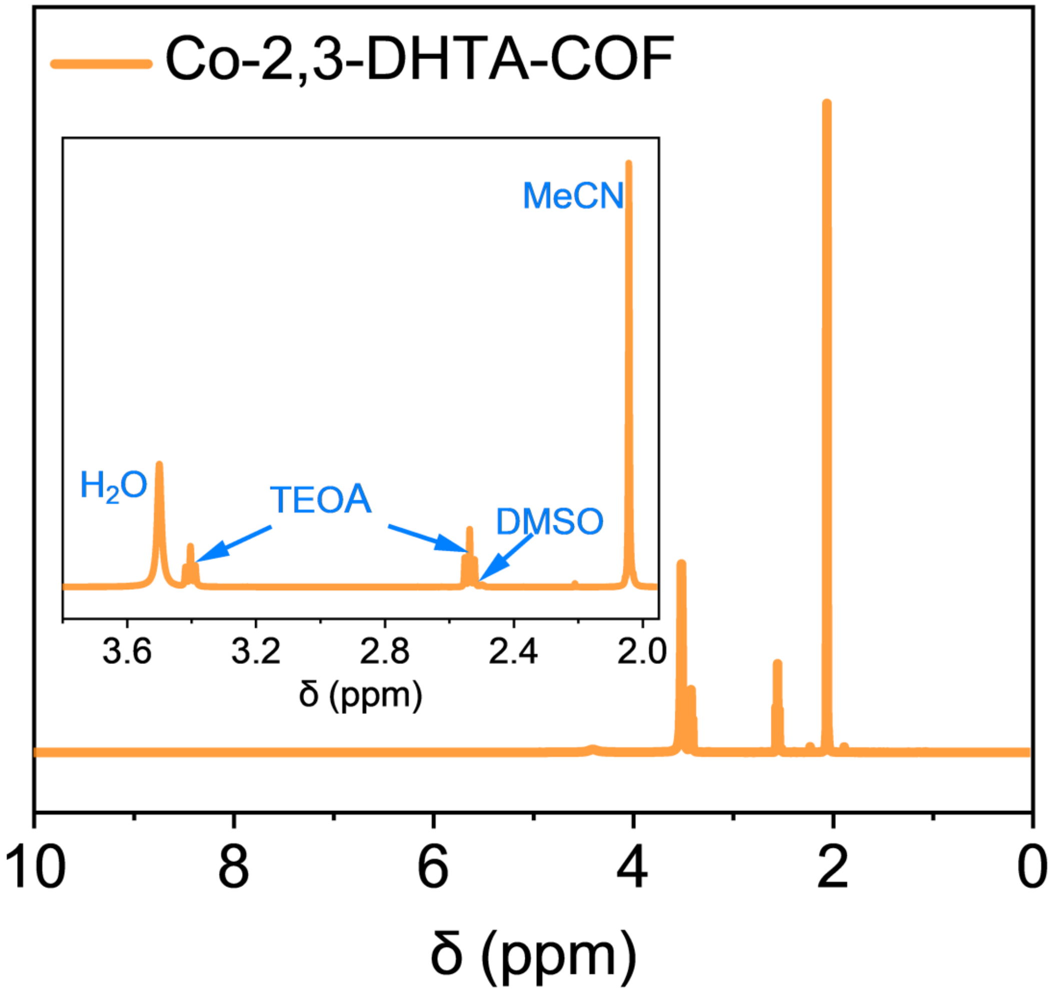

Supplementary Figure 20 ^{1}H-NMR spectra. ^{1}H-NMR spectra of the liquid products after a 4 h CO_2 reduction catalyzed by optimal Co-2,3-DHTA-COF. d^6-DMSO was used as the internal standard.

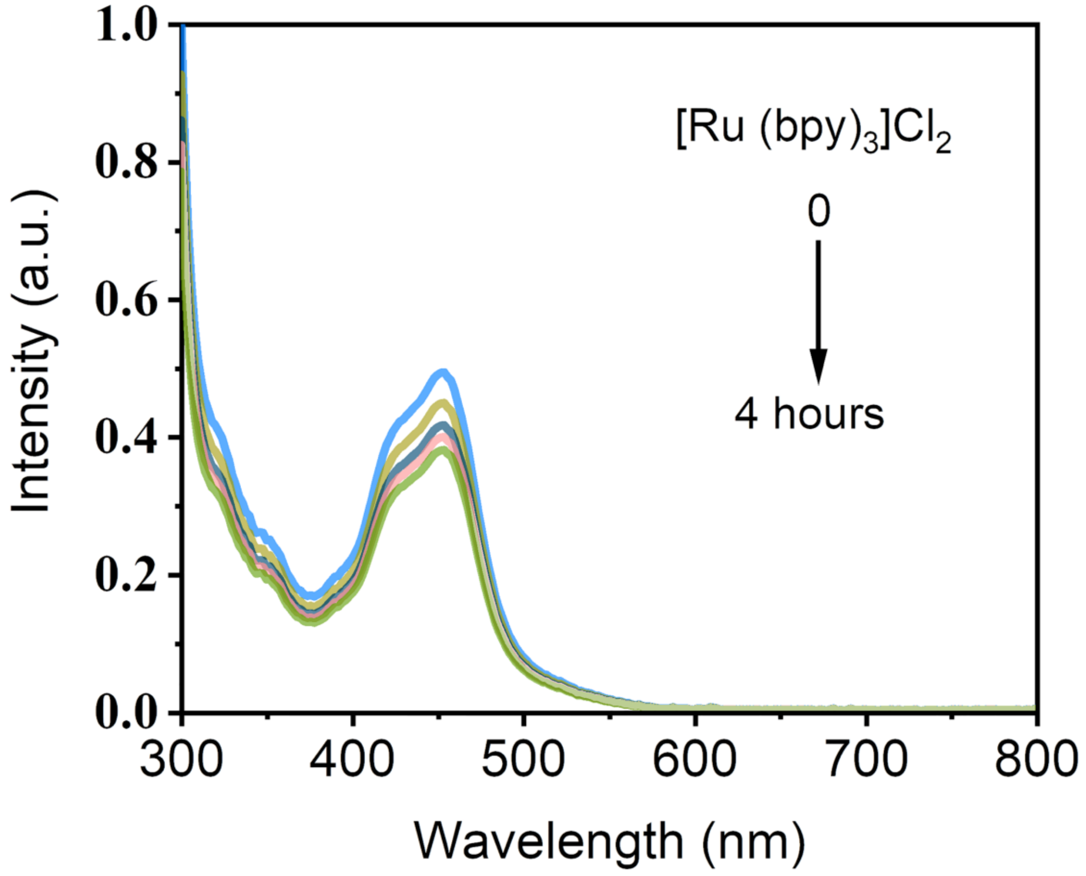

Supplementary Figure 21 UV-vis absorption spectra of $[Ru(bpy)_3]Cl_2$. UV-vis absorption spectra of photosensitizer $[Ru(bpy)_3]Cl_2$ as a function of time.

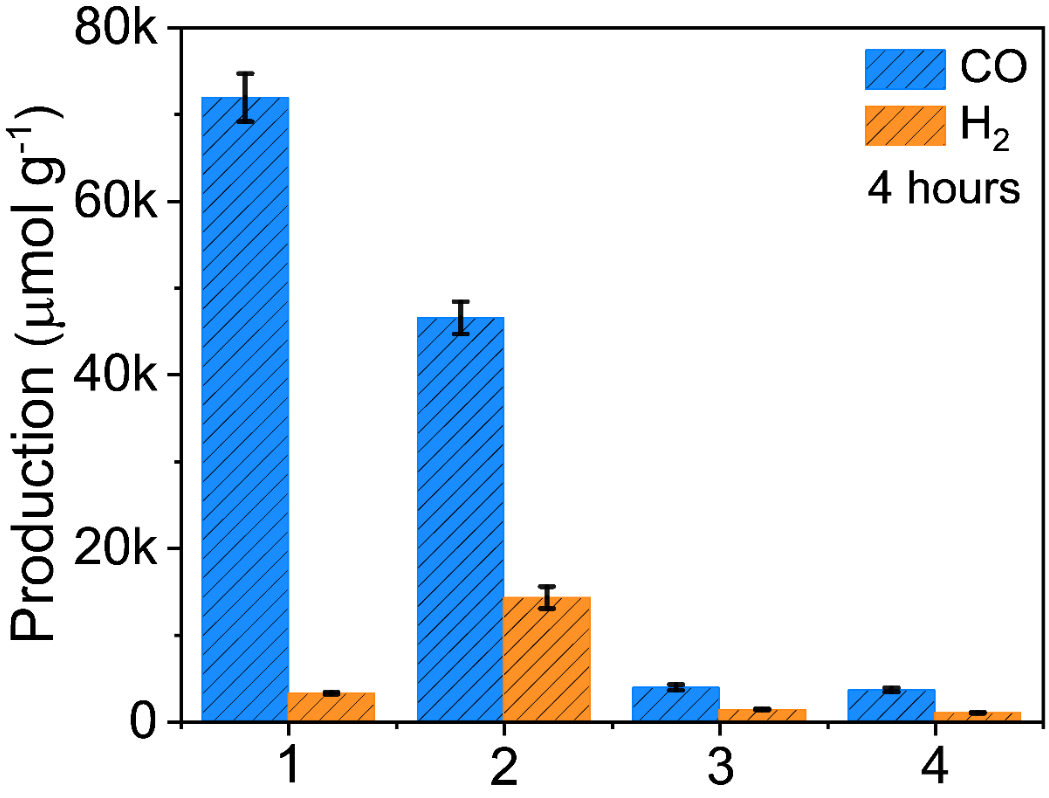

Supplementary Figure 22 CO_2 photoreduction performance catalyzed by neat COF and Co-COF (test duration, 4 hours). 1, Co-2,3-DHTA-COF. **2**, Co-TP-COF. **3**, 2,3-DHTA-COF. **4**, TP-COF. The error bars for CO production uncertainty represent one standard deviation based on three independent samples.

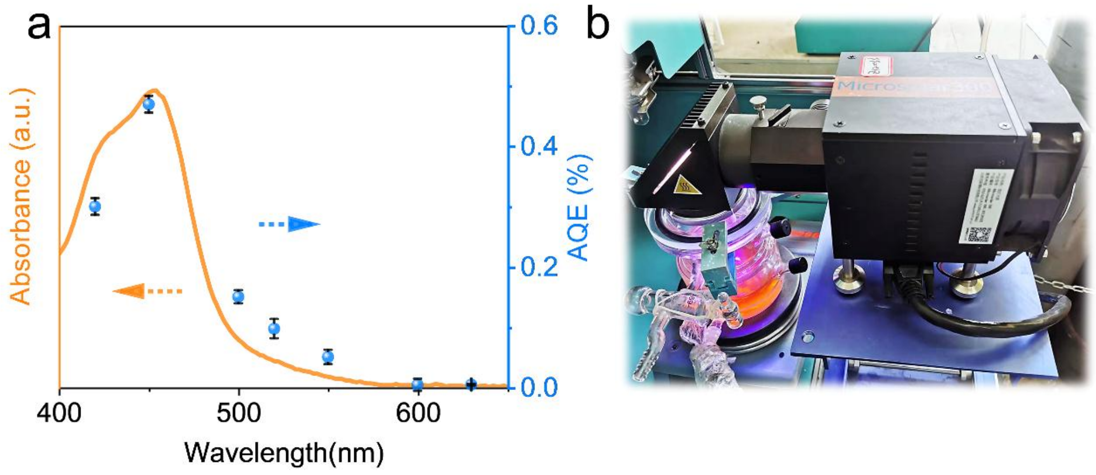

Supplementary Figure 23 AQE of CO production catalyzed by **a** Co-2,3-DHTA-COF at different wavelengths and the related visible spectra. **b** The digital image of the measurement device. The error bars for AQE uncertainty represent one standard deviation based on three independent samples.

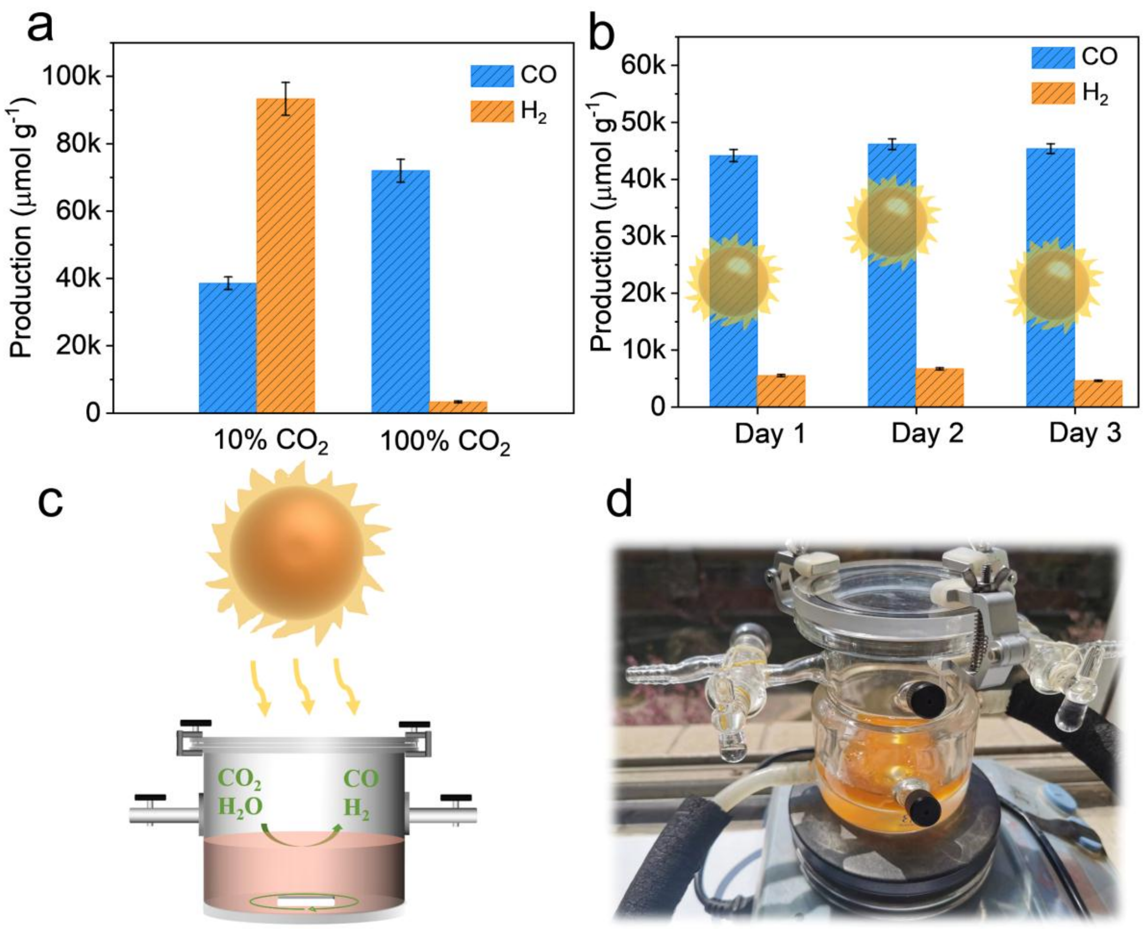

Supplementary Figure 24 photoreduction performance. **a** Comparison of CO_2 photoreduction performance under the condition of 10 vol% CO_2 and pure CO_2 atmosphere for 4-hour conversion catalyzed by Co-2,3-DHTA-COF. **b** Performance tested under natural sunlight (test duration: 4 hours per day). **c, d** Scheme and photo image of the practical device used for CO_2 photocatalytic reduction under sun light. The error bars for CO production uncertainty represent one standard deviation based on three independent samples.

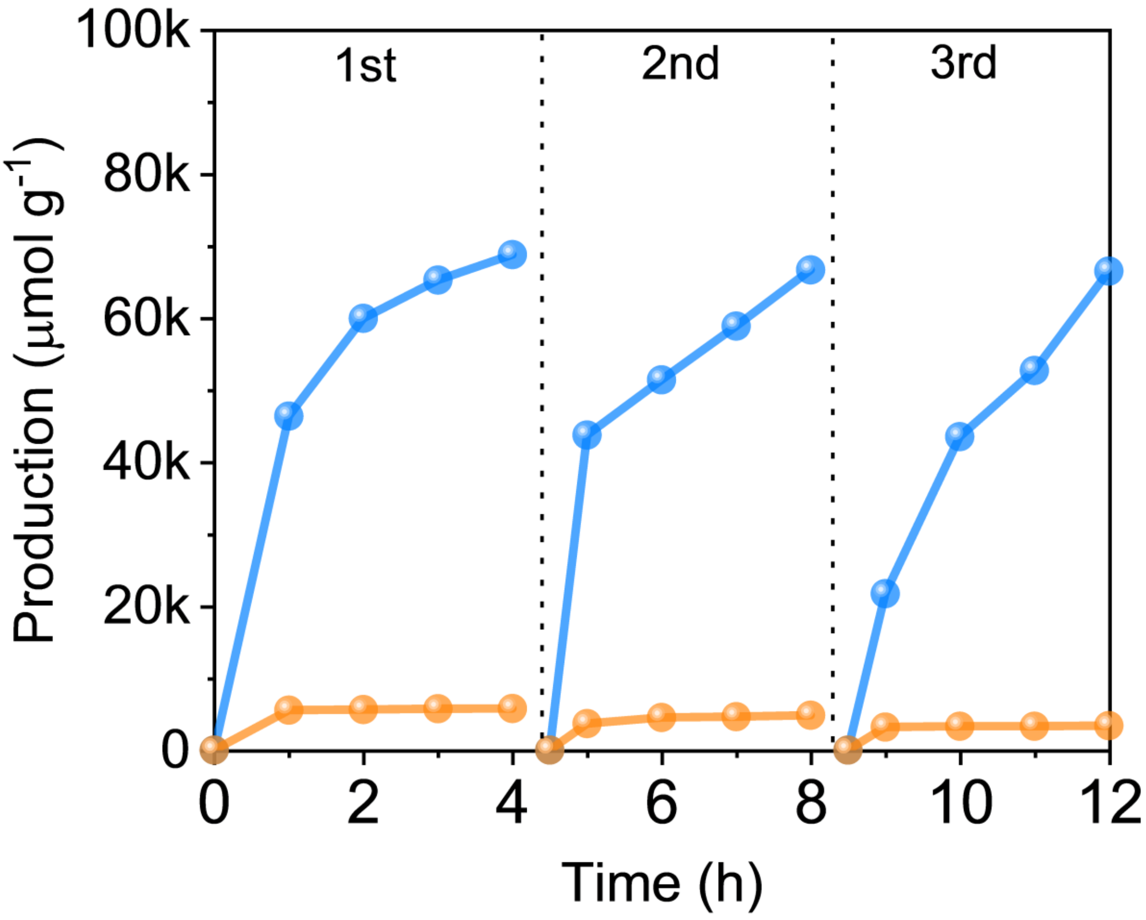

Supplementary Figure 25 Photocatalytic stability of Co-2,3-DHTA-COF. Stability of Co-2,3-DHTA-COF during 3 cycles of CO_2 photocatalytic reduction process to CO (blue) and H_2 (orange).

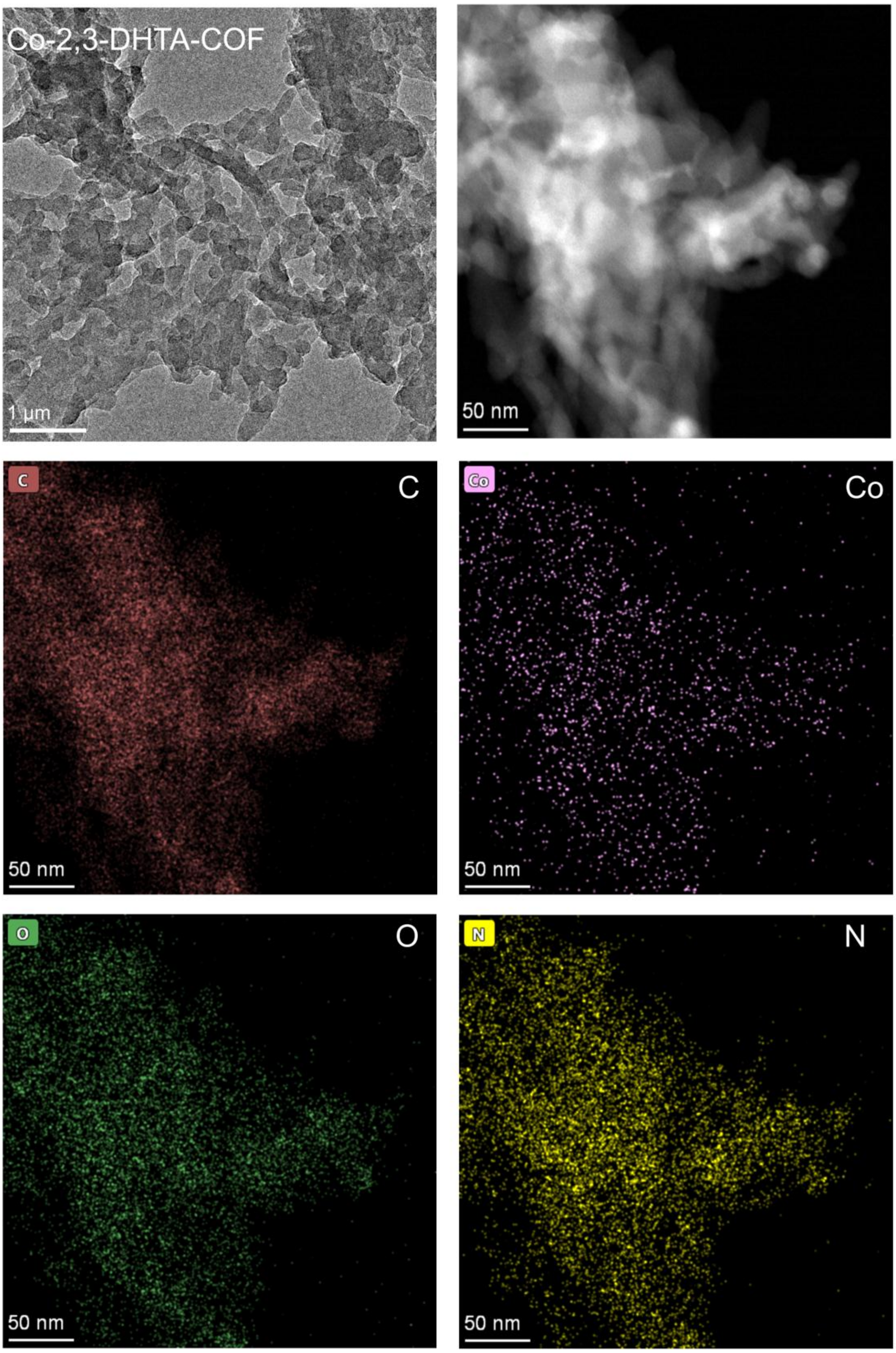

Supplementary Figure 26 TEM images and corresponding elemental mapping after photocatalysis. TEM images and corresponding elemental mapping of Co-2,3-DHTA-COF after 12 hours photocatalytic tests.

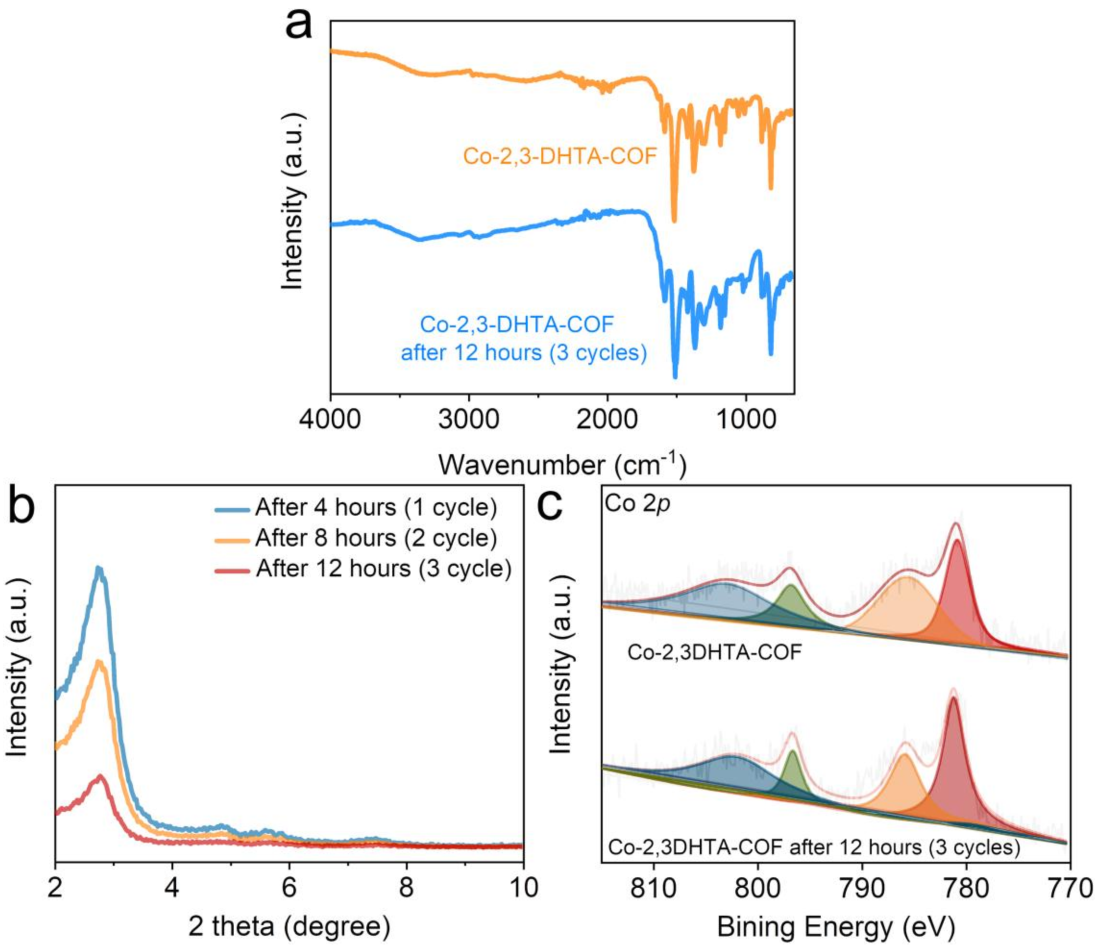

Supplementary Figure 27 Chemical stability of Co-2,3-DHTA-COF before and after 12 hours CO_2 photoreduction process. a FTIR spectra. **b** XRD patterns. **c** High-resolution XPS spectra.

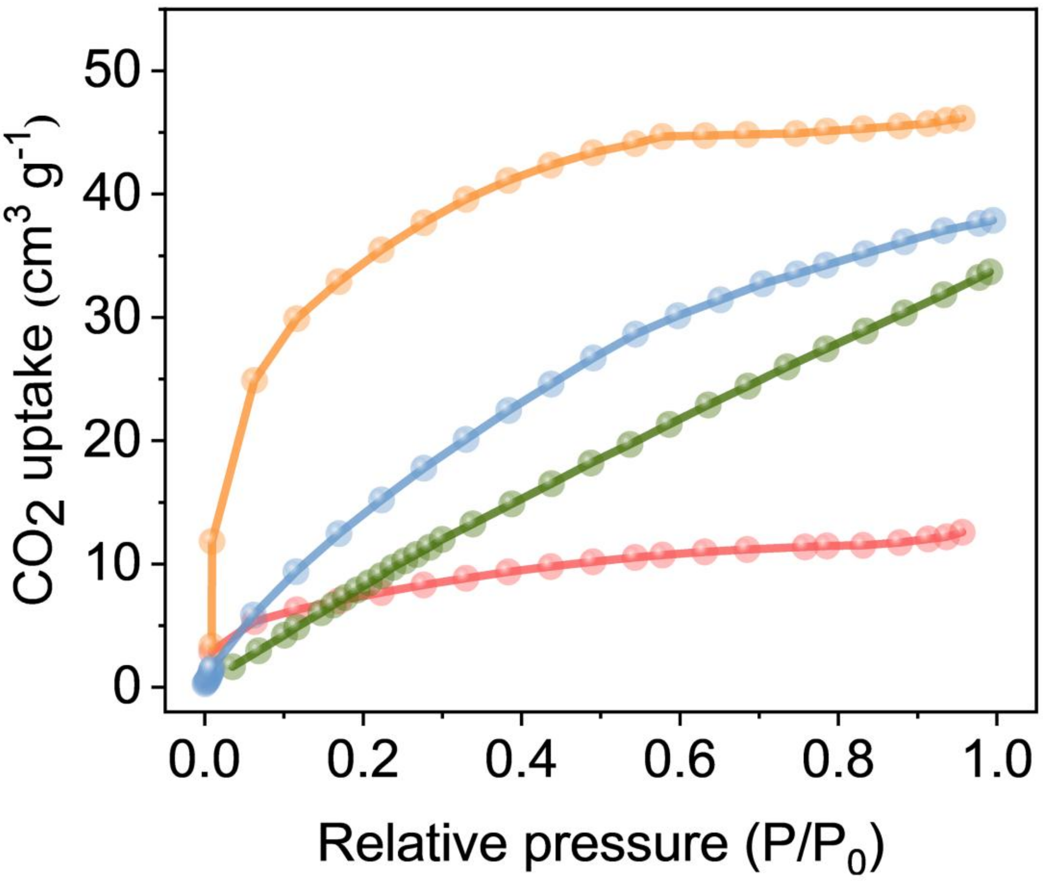

Supplementary Figure 28 CO_2 adsorption of all the samples. CO_2 adsorption isotherms on Co-2,3-DHTA-COF (orange), Co-TP-COF (blue), TP-COF (green), and 2,3-DHTA-COF (red) at 25 °C.

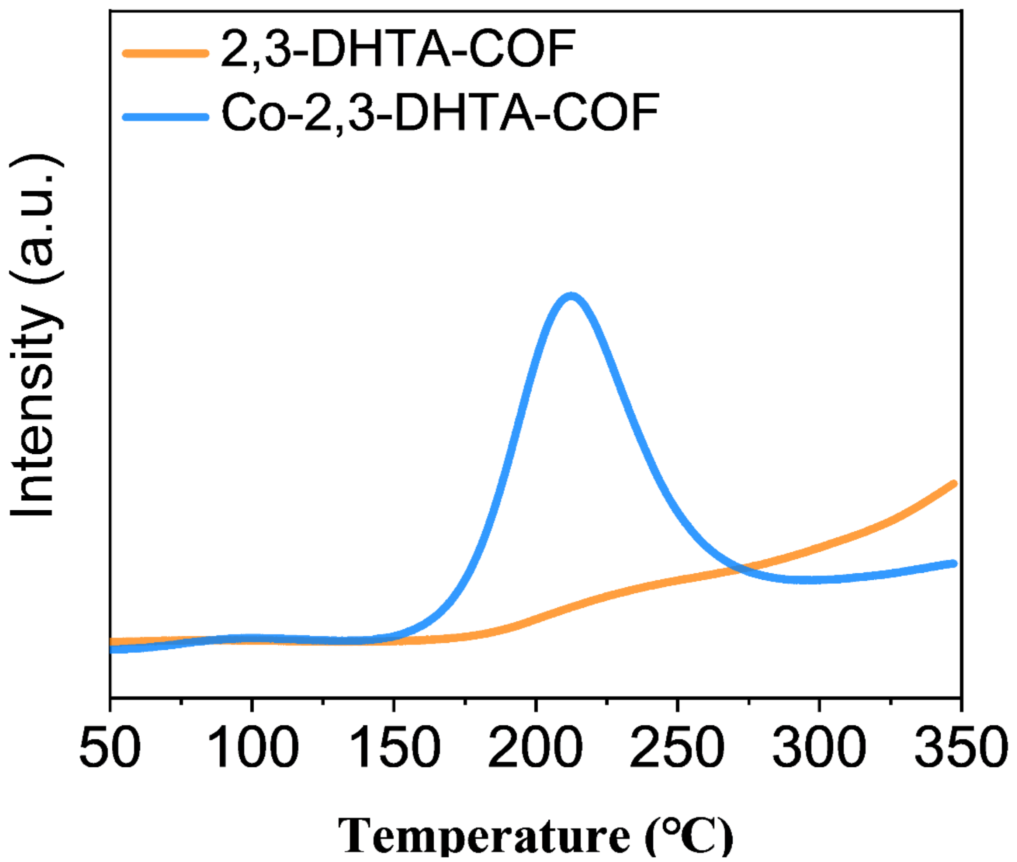

Supplementary Figure 29 CO_2 temperature-programmed desorption curve (CO_2-TPD). CO_2-TPD curve of Co-2,3-DHTA-COF (2.14 wt.%) and 2,3-DHTA-COF.

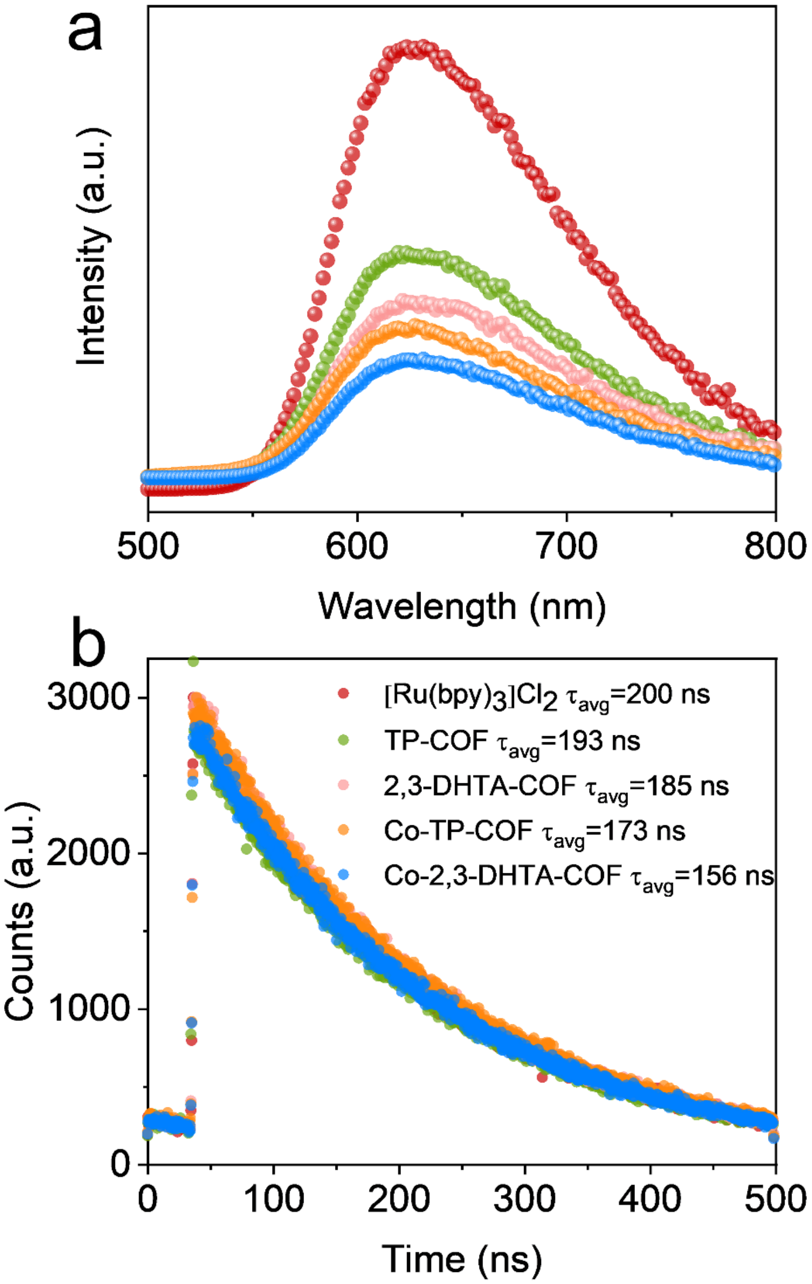

Supplementary Figure 30 Steady-state PL spectra and PL decay spectra of Co-COF/COF in reaction system. a Steady-state PL spectra of [Ru $(bpy)_3$]Cl_2 (red), TP-COF (green), 2,3-DHTA-COF (pink), Co-TP-COF (orange), and Co-2,3-DHTA-COF (blue). **b** PL decay spectra of the CO_2 photoreduction system with ([Ru $(bpy)_3$]Cl_2+COF or Co-COF) and without (only [Ru $(bpy)_3$]Cl_2) addition of COF or Co-COF).

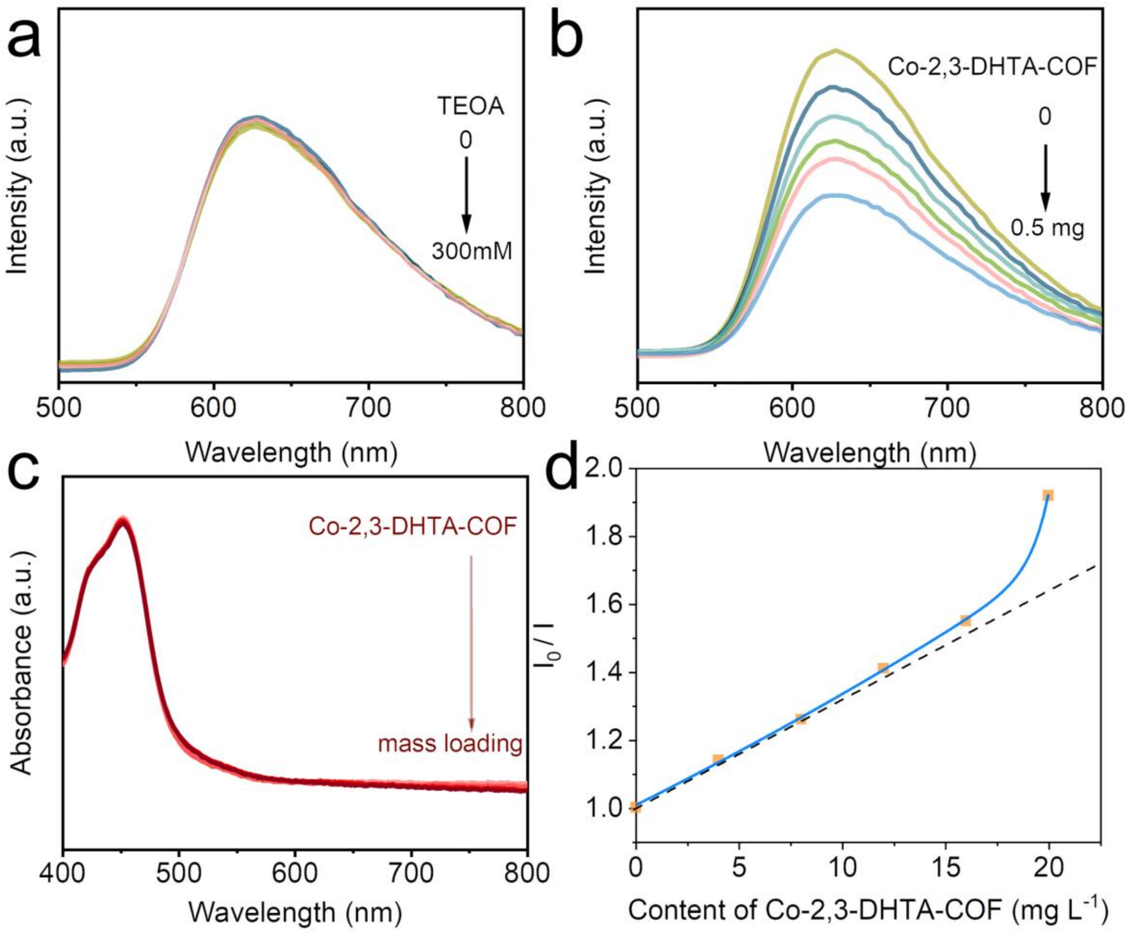

Supplementary Figure 31 Steady-state PL spectra and visible absorption spectra of Co-2,3-DHTA-COF. a Steady-state PL spectra of the CO_2 photoreduction system with the addition of TEOA. **b** Visible absorption spectra of the aqueous solution containing $[Ru(bpy)_3]Cl_2$ and different amounts of Co-2,3-DHTA-COF (0, 0.10, 0.20, 0.30, 0.40, 0.50 mg). **c** Steady-state PL spectra of the CO_2 photoreduction system with the addition of Co-2,3-DHTA-COF. **d** The plot of the steady-state PL intensities of $[Ru(bpy)_3]Cl_2$ versus the content of Co-2,3-DHTA-COF, fitting with the Stern−Volmer equation (blue line). The dotted line refers to collision quenching.

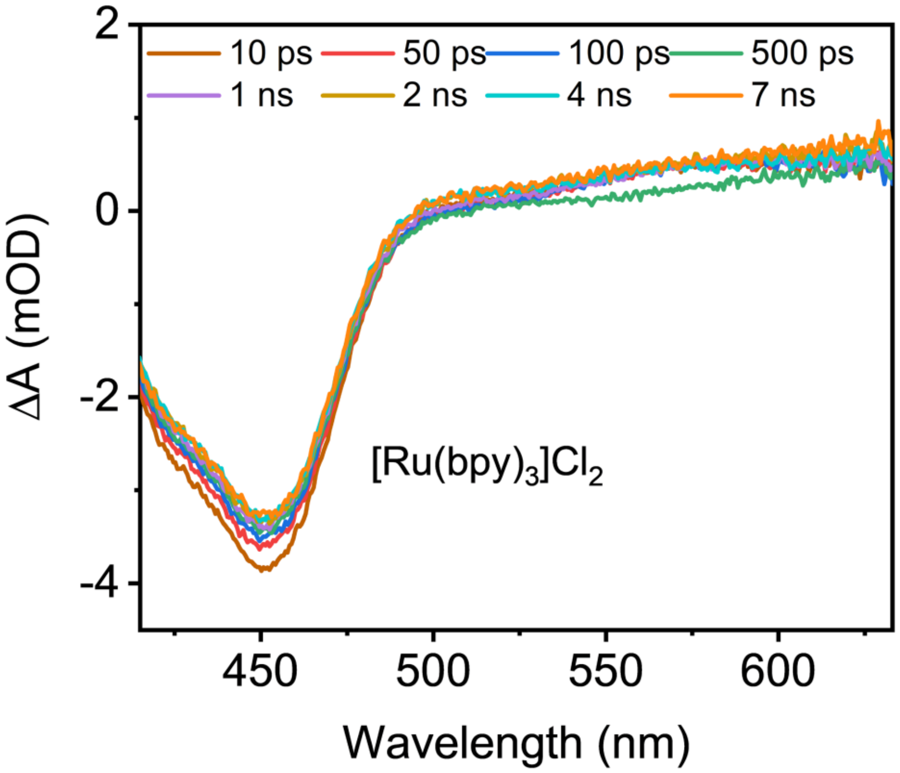

Supplementary Figure 32 Transient absorption spectroscopy (fs-TA). The fs-TA spectra of $[Ru(bpy)_3]Cl_2$ from 370 nm to 650 nm (excited at 365 nm).

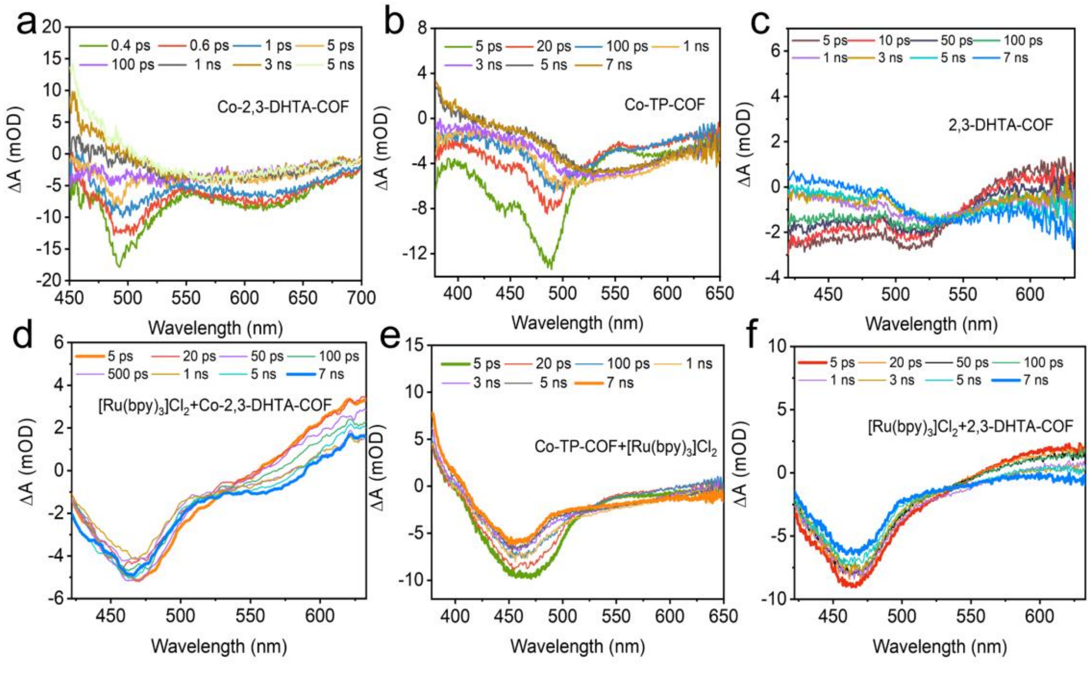

Supplementary Figure 33 Transient absorption spectroscopy (fs-TA) in the probe wavelength range from 370 nm to 650 nm (excited at 365 nm). a Co-2,3-DHTA-COF. **b** Co-TP-COF. **c** 2,3-DHTA-COF. **d** $[Ru(bpy)_3]Cl_2$+Co-2,3-DHTA-COF. **e** $[Ru(bpy)_3]Cl_2$ +Co-TP-COF. **f** $[Ru(bpy)_3]Cl_2$+2,3-DHTA-COF.

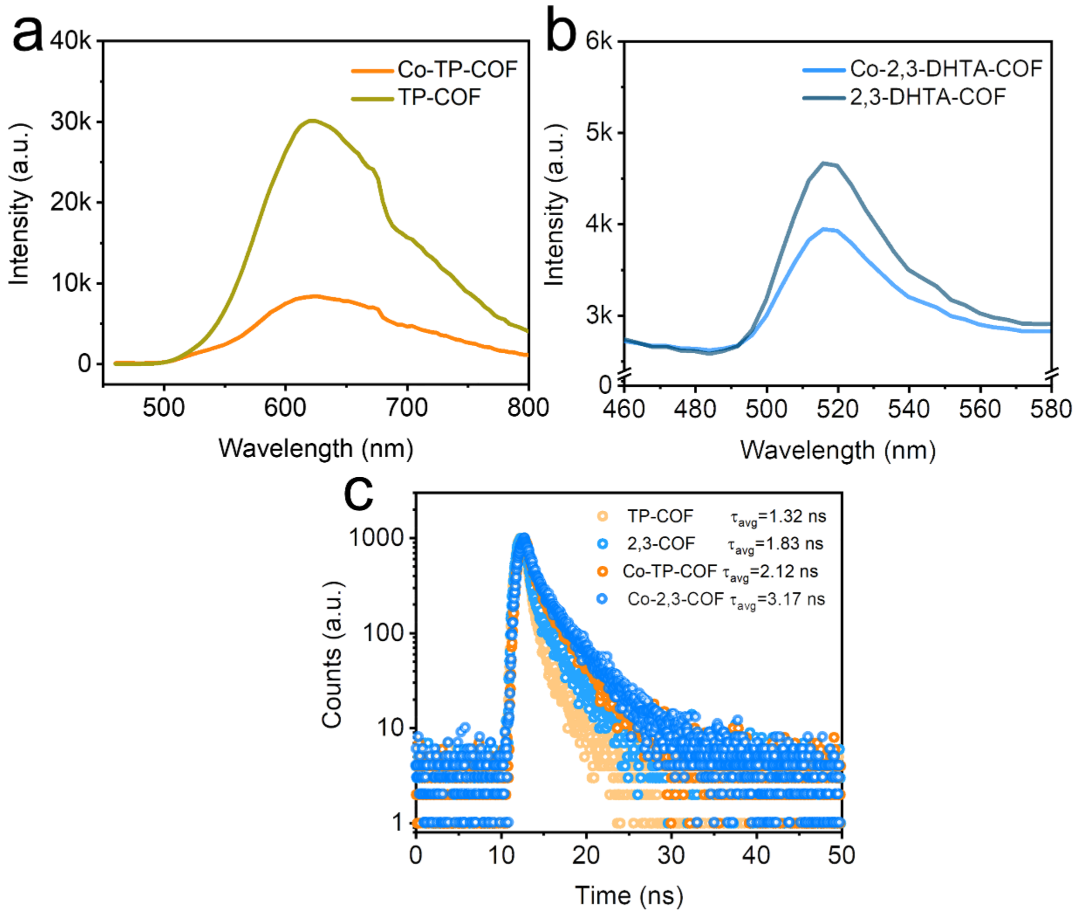

Supplementary Figure 34 Steady-state PL spectra and PL decay spectra of Co-COF/COF powder. Steady-state PL spectra of **a** TP -COF and Co-TP –COF, **b** 2,3-DHTA-COF and Co-2,3-DHTA-COF. **c** PL decay spectra of TP -COF, 2,3-DHTA-COF, Co-TP -COF and Co-2,3-DHTA-COF.

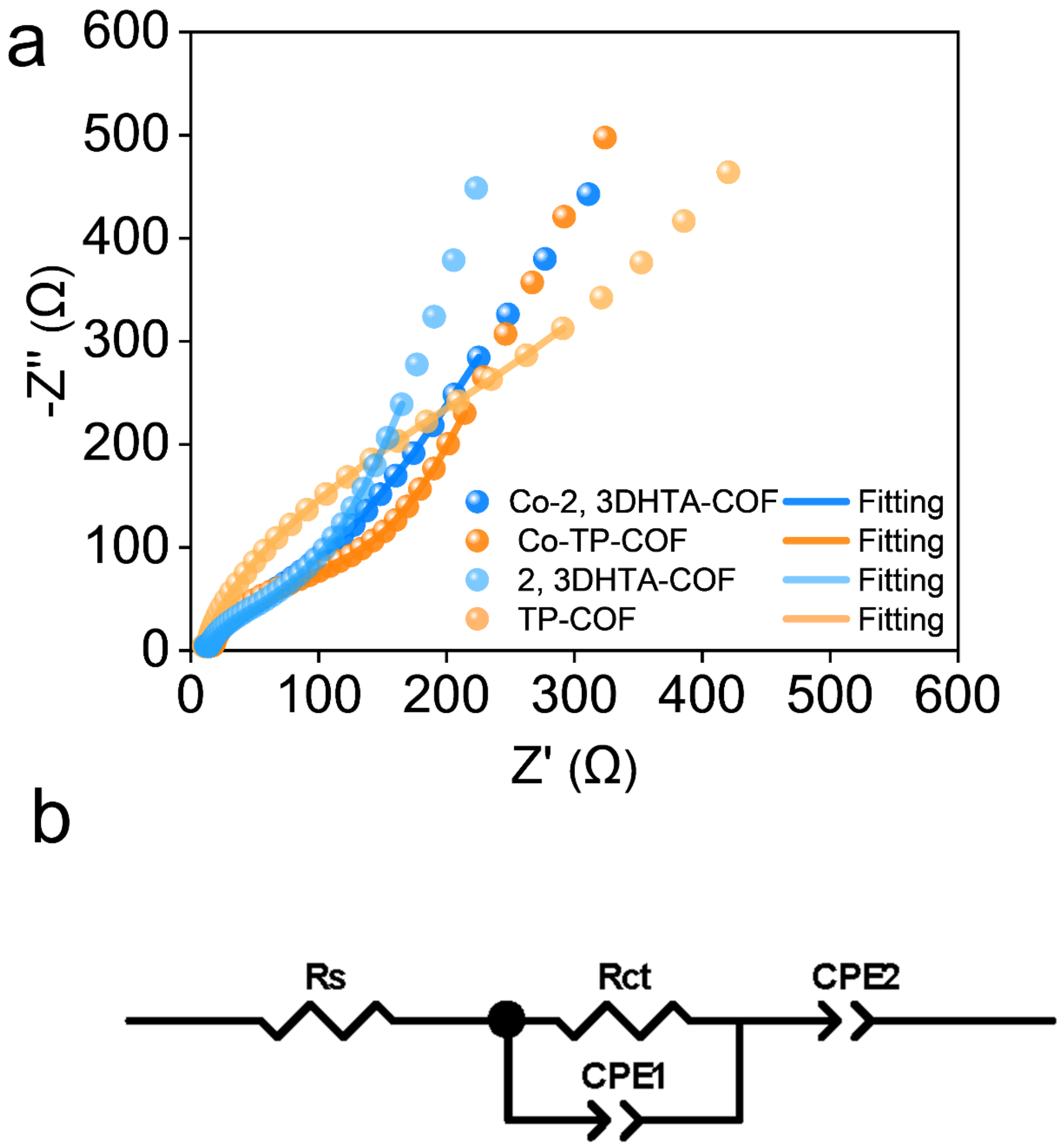

Supplementary Figure 35 Nyquist plots of all the samples based on electrochemical impedance spectroscopy (EIS) measurements. a Co-COF/COF. **b** The related equivalent circuit.

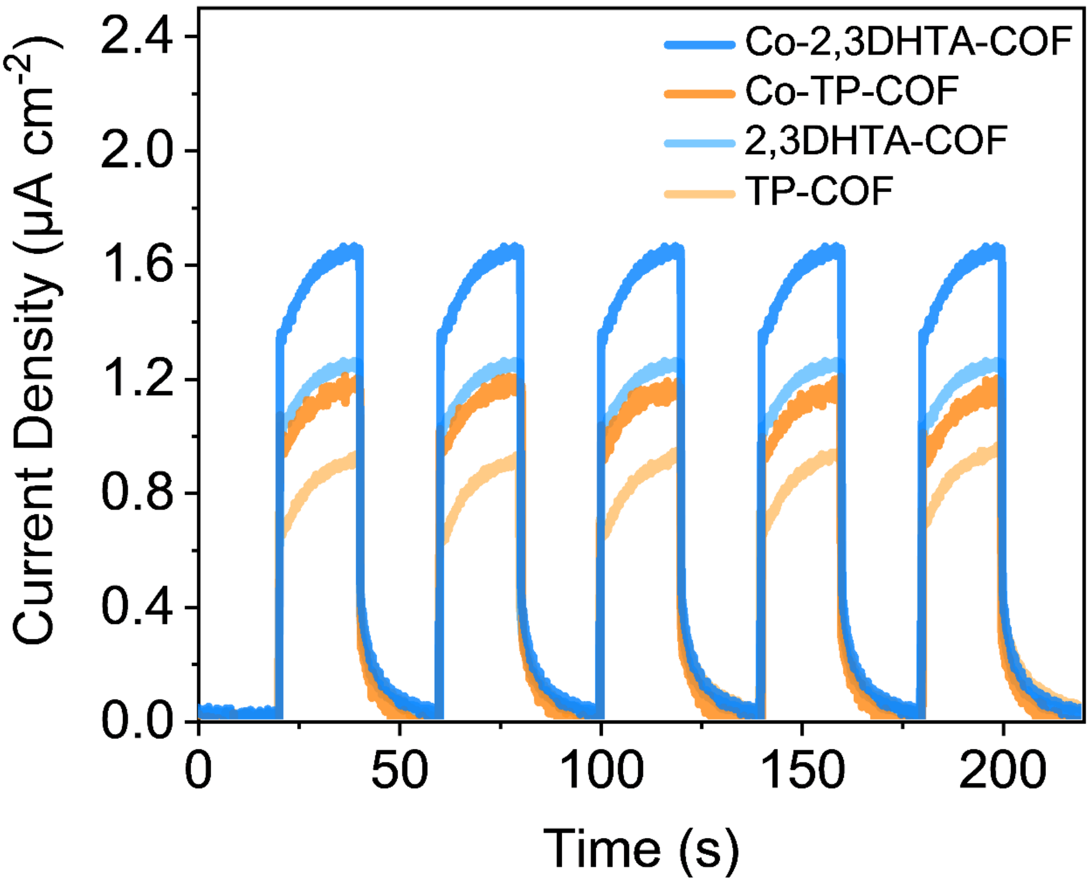

Supplementary Figure 36 Photoresponsive current density analyst. Transient photocurrent density under Xenon lamp (≥ 420 nm) irradiation for all the samples.

3. Supplementary Tables 1-8

Supplementary Table 1. Cobalt content in Co-2,3DHTA-COF and Co-TP-COF determined by ICP-MS and XPS

Sample	Metal	ICP-Content (wt.%)	XPS-Atomic (wt.%)
Co-2,3-DHTA-COF	Co	2.14	2.42
Co-TP-COF	Co	1.05	1.71

Supplementary Table 2. EXAFS fitting results of Co-2,3-DHTA-COF and Co-TP-COF[a]

Sample	Shell	CN	R(Å)	σ^2(Å^2)	ΔE_0(eV)	*R factor*
Cobalt foil	Co-Co	12*	2.49±0.01	0.0061±0.0004	5.9±0.595	0.004
CoPc	Co-N	4.0	1.89±0.01	0.002±0.002	3.50±3.23	0.018
CoO	Co-O	6.0±0.9	2.12±0.01	0.01±0.0041	0.16±1.8	0.004
	Co-Co	12.6±1.2	3.00±0.01	0.009±0.0010	-2.46±0.9	
Co_3O_4	Co-O	5.2±0.5	1.91±0.01	0.0025±0.0009	-6.5±1.5	0.006
	Co-Co	3.9±1.1	2.84±0.01	0.0027±0.0015	-7.5±2.5	
	Co-Co	11.7±3.2	3.35±0.01	0.0079±0.002	-7.8±1.8	
Co-2,3-DHTA-COF	Co-O	4.15±0.26	2.09±0.01	0.01±0.0009	0.29±0.67	0.003
Co-TP-COF	Co-N/O	3.98±0.34	2.03±0.01	0.009±0.0014	7.14±1.01	0.009

[a]Data Range: Δk, 3-12.5 Å^{-1}; ΔR, 1-2.5 Å; K weight =3.0
CN = coordination number, R = interatomic distance (the bond length between central atoms and surrounding coordination atoms) based on fitting results, ΔE_0 = inner potential correction, σ^2 = Debye-Waller factor, and the *R factor* is used to value the goodness of fit.

Supplementary Table 3. Production rate and selectivity of CO_2RR to CO catalyzed by different photocatalysts

Photocatalyst	Production rate of CO (μmol g^{-1} h^{-1})	Production rate of H_2 (μmol g^{-1} h^{-1})	CO selectively (%)
Co-2,3-DHTA-COF	18000	800	95.7
Co-TP-COF	11600	3560	76.6
2,3-DHTA-COF	760	230	76.7
TP-COF	960	330	74.5

Supplementary Table 4. The CO production rate and the mass of Co-2,3-DHTA-COF used in five independent experiments

Catalyst mass (mg)	CO production rate (μmol g^{-1} h^{-1})
1.01	17980
1.02	17995
1.05	17860
1.01	17960
1.03	17987

Supplementary Table 5. Comparison of CO_2 photoreduction performance catalyzed by various metal-supported porous organic materials

Photocatalyst	PS	Source	SA	Light Condition	Production rate for CO (μmol g^{-1} h^{-1})	CO selectivity	References
Co-2,3-DHTA-COF	$[Ru(bpy)_3]Cl_2 \cdot 6H_2O$	$MeCN/H_2O$ (v/v=4/1)	TEOA	300 W Xe lamp (Cut 420 nm)	18000±700	95.7%	This Work
COF-367-Co NSs	$[Ru(bpy)_3]Cl_2 \cdot 6H_2O$	0.1 M $KHCO_3$	Ascorbic Acid	300 W Xe lamp (Cut 420 nm)	10162	78%	14
Co-FPy-CON	$(Ir[dF(CF_3)ppy]_2(dtbpy))PF_6$	$MeCN/H_2O$ (v/v=1/1)	TEOA	300 W Xe lamp (Cut 420 nm)	1683	76%	15
DQTP COF-Co	$[Ru(bpy)_3]Cl_2 \cdot 6H_2O$	MeCN	TEOA	300 W Xe lamp (Cut 420 nm)	1020	90%	16
NiPc-CoPOP	$[Ru(bpy)_3]Cl_2 \cdot 6H_2O$	$MeCN/H_2O$ (v/v=16/1)	TEOA	White LED (400 nm-800 nm)	4270	54%	17
TFBD-COF-Co-SA	$[Ru(bpy)_3]Cl_2 \cdot 6H_2O$	MeCN	TEOA	300 W Xe lamp (Cut 400 nm)	1480	90%	18
rGO$_{15}$@TpPa-1	2’2-bipyridine $CoCl_2$	MeCN	TEOA	300 W Xe lamp (Cut 420 nm)	200	89%	19
C_3N_4 (NH)/COF	2’2-bipyridine (bpy), $CoCl_2$	$MeCN/H_2O$	TEOA	300 W Xe lamp (Cut 400 nm)	562	90%	20
CTF-TDPN	$Co(OAc)_2$ tetrahydrate 2,2'-Bipyridine	$MeCN/H_2O$ (v/v=3/1)	TEOA	300 W Xe lamp (Cut 420 nm)	190	58%	21
Co@COP-30	/	$MeCN/H_2O$ (v/v=3/1)	TEOA	300 W Xe lamp (Cut 400 nm)	506	94%	22
NiP-TPE-COF	$[Ru(bpy)_3]Cl_2 \cdot 6H_2O$	MeCN	TEOA	300 W Xe lamp (Cut 420 nm)	525	93%	23
Ni-TpBpy	$[Ru(bpy)_3]Cl_2 \cdot 6H_2O$ 2,2′-bipyridine	$MeCN/H_2O$ (v/v=3/1)	TEOA	300 W Xe lamp (Cut 420 nm)	4057	96%	24
NAHN-Tp COF	[Ir-ppy]	MeCN	TEOA	300 W Xe lamp (Cut 420 nm)	8.86	98%	25
Ni-PCD @ TD-COF	$[Ru(bpy)_3]Cl_2 \cdot 6H_2O$	$MeCN/H_2O$ (v/v=3/1)	TEOA	300 W Xe lamp (Cut 420 nm)	480	98%	26
PI-COFs	2,2'-Bipyridine $Ni(ClO4)_2$ $6H_2O$	$MeCN/H_2O$ (v/v=3/1)	TEOA	300 W Xe lamp (Cut 420 nm)	480	93%	27
H-COF-Ni	$[Ru(bpy)_3]Cl_2 \cdot 6H_2O$ 2,2′-bipyridine	$MeCN/H_2O$ (v/v=3/1)	TEOA	300 W Xe lamp (Cut 420 nm)	5694	96%	28
Ni@TPHH-COF	$[Ru(bpy)_3]Cl_2 \cdot 6H_2O$	$MeCN/H_2O$ (v/v=4/1)	TEOA	300 W Xe lamp (Cut 420 nm)	1610	96%	29
Re-Bpy-sp^{2}c-COF	/	MeCN	TEOA	300 W Xe lamp (Cut 420 nm)	1040	81%	30
α-Fe_2O_3 @Por-CTF	$[Ru(bpy)_3]Cl_2 \cdot 6H_2O$	DMF	TEOA	300 W Xe lamp (Cut 420 nm)	400	93%	4
NiPc-NiPOP	$[Ru(bpy)_3]Cl_2 \cdot 6H_2O$	$MeCN/H_2O$ (v/v=9/1)	TEOA	White LED (400 nm-800 nm)	1940	96%	17
CdS@COF	/	$MeCN/H_2O$ (v/v=4/1)	BIH	300 W Xe lamp (Cut 420 nm)	507	72%	31
HOF-25-Re	$[Ru(bpy)_3]Cl_2 \cdot 6H_2O$	MeCN	TIPA	300 W Xe lamp (Cut 420 nm)	3030	92%	32

Supplementary Table 6. Apparent quantum efficiency (AQE) of Co-2,3-DHTA-COF at different wavelengths

Wavelengths (nm)	Intensity (mW cm^{-2})	AQE (%)
420	14.6	0.300
450	16.3	0.470
500	16.0	0.140
520	18.2	0.097
550	18.5	0.050
600	20.2	0.004
630	21.5	0.004

Supplementary Table 7. Average temperature and sunlight intensity during the outdoor tests

Day	Temperature (°C)	Light intensity (mW cm^{-2})
1	18	41.5
2	19	43.6
3	21	48.7

Supplementary Table 8. Charge transfer resistance (R_{ct}) of all the samples

Sample	R_{ct}/Ω
Co-2,3-DHTA-COF	23
Co-TP-COF	42
2,3-DHTA-COF	158
TP-COF	109

4. Supplementary References

1 Lu, M. et al. Confining and highly dispersing single polyoxometalate clusters in covalent organic frameworks by covalent linkages for CO_2 photoreduction. *J. Am. Chem. Soc.* **144**, 1861-1874 (2022).

2 Wang, S., Yao, W. S., Lin, J. L., Wang, X. C. Cobalt imidazolate metal–organic frameworks photosplit CO_2 under mild reaction conditions. *Angew. Chem. Int. Ed.* **53**, 1034-1038 (2014).

3 Zhao, W. et al. Unblocked intramolecular charge transfer for enhanced CO_2 photoreduction enabled by an imidazolium-based ionic conjugated microporous polymer. *Appl. Catal. B: Environ.* **300**, 120719 (2022).

4 Zhang, S. et al. An artificial photosynthesis system comprising a covalent triazine framework as an electron relay facilitator for photochemical carbon dioxide reduction. *J. Mater. Chem. C.* **8**, 192 (2020).

5 Jiang, Z. et al. Filling metal–organic framework mesopores with TiO_2 for CO_2 photoreduction. *Nature*, **586**, 549-554 (2020).

6 Lam, E., Reisner, E. A TiO_2-Co(terpyridine)$_2$ photocatalyst for the selective oxidation of cellulose to formate coupled to the reduction of CO_2 to syngas. *Angew. Chem. Int. Ed.* **60**, 23306-23312 (2021).

7 Lee, C. et al. Development of the colle-salvetti correlation-energy formula into a functional of the electron density. *Physical. Review. B.* **37**, 785-789 (1988).

8 Becke, A. D. Density‐functional thermochemistry. III. the role of exact exchange. *J. Chem. Phys.* **98**, 5648-5652 (1993).

9 Hay, P. J., Wadt, W. R. Ab initio effective core potentials for molecular calculations. Potentials for K to Au including the outermost core orbitals. *J. Chem. Phys.* **82**, 299-310 (1985).

10 Francl, M. M. et al. Self‐consistent molecular orbital methods. XXIII. A polarization‐type basis set for second‐row elements. *J. Chem. Phys.* **77**, 3654-3665 (1982).

11 Cancès, E., Mennucci, B., Tomasi, J. A new integral equation formalism for the polarizable continuum model: theoretical background and applications to isotropic and anisotropic dielectrics. *J. Chem. Phys.* **107**, 3032-3041 (1997).

12 Frisch, M. R., Trucks, G. W., Schlegel, H. B., Scuseria, G. E., Robb, M. A., Cheeseman, J. R., Scalmani, G., Barone, V., Petersson, G. A., Nakatsuji, H., Li, X., Caricato, M., Marenich, A. V., Bloino, J., Janesko, B. G., Gomperts, R., Mennucci, B., Hratchian, H. P., Ortiz, J. V., Izmaylov, A. F.,

Sonnenberg, J. L., Williams-Young, D., Ding, F., Lipparini, F., Egidi, F., Goings, J., Peng, B., Petrone, A., Henderson, T., Ranasinghe, D., Zakrzewski, V. G., Gao, J., Rega, N., Zheng, G., Liang, W., Hada, M., Ehara, M., Toyota, K., Fukuda, R., Hasegawa, J., Ishida, M., Nakajima, T., Honda, Y., Kitao, O., Nakai, H., Vreven, T., Throssell, K., Montgomery, J. A., Jr., Peralta, J. E., Ogliaro, F., Bearpark, M. J., Heyd, J. J., Brothers, E. N., Kudin, K. N., Staroverov, V. N., Keith, T. A., Kobayashi, R., Normand, J., Raghavachari, K., Rendell, A. P., Burant, J. C., Iyengar, S. S., Tomasi, J., Cossi, M., Millam, J. M., Klene, M., Adamo, C., Cammi, R., Ochterski, J. W. Martin, R. L., Morokuma, K., Farkas, O., Foresman, J. B., Fox, D. J., Gaussian 16, Gaussian, Inc.: Wallingford, CT. (2016)

13 Marenich, A. V., Ho, J., Cote, M. L., Cramer, C. J., Truhlar, D. G. Computational electrochemistry: prediction of liquid-phase reduction potentials. *Phys. Chem. Chem. Phys.* **16**, 15068-15106 (2014).

14 Liu, W. et al. A scalable general synthetic approach toward ultrathin imine-linked two-dimensional covalent organic framework nanosheets for photocatalytic CO_2 reduction. *J. Am. Chem. Soc.* **141**, 17431-17440 (2019).

15 Wang, X. et al. Covalent organic framework nanosheets embedding single cobalt sites for photocatalytic reduction of carbon dioxide. *Chem. Mater.* **32**, 9107-9114 (2018).

16 Lu, M. et al. Installing earth-abundant metal active centers to covalent organic frameworks for efficient heterogeneous photocatalytic CO_2 reduction. *Appl. Catal. B: Environ.* **254**, 624-633 (2019).

17 Dong, X. Y., Si, Y. N., Wang, S., Zang, S. Q. Integrating single atoms with different microenvironments into one porous organic polymer for efficient photocatalytic CO_2 reduction. *Adv. Mater.* **33**, 2101568 (2021).

18 Yang, Y. et al. Decoration of active sites in covalent-organic framework: an effective strategy of building efficient photocatalysis for CO_2 reduction. *ACS Sustainable. Chem. Eng.* **9**, 13376 (2021).

19 Gopalakrishnan, V. N. et al. Manifestation of an enhanced photoreduction of CO_2 to CO over the in situ synthesized rGO–covalent organic framework under visible light irradiation. *ACS Appl. Energy. Mater.* **4**, 6005-6014 (2021).

20 Wang, J. et al. Defective g-C_3N_4/covalent organic framework van der Waals heterojunction toward highly efficient S-scheme CO_2 photoreduction. *Appl. Catal. B: Environ.* **301**, 120814 (2022).

21 He, Y. et al. Encapsulation of Co single sites in covalent triazine frameworks for photocatalytic production of syngas. *Chinese J. Catal.* **42**, 123-130 (2021).

22 Chen, J. Q., Zhong, H., Lv, H. W., Liu, R. X., Wang, R. H. Regulating utilization efficiency of the photogenerated charge carriers by constructing donor–π–acceptor polymers for upgrading photocatalytic CO_2 reduction. *ChemSusChem.* **14**, 2749-2756 (2021).

23 Lv, H. et al. Metalloporphyrin-based covalent organic frameworks composed of the electron donor-acceptor dyads for visible-light-driven selective CO_2 reduction. *Sci. China Chem.* **63**, 1289-1294 (2020).

24 Zhong, W. et al. A covalent organic framework bearing single Ni sites as a synergistic photocatalyst for selective photoreduction of CO_2 to CO. *J. Am. Chem. Soc.* **141**, 7615-7621 (2019).

25 You, S. Q. et al. A hydrazone-based covalent organic framework/iridium (III) complex for photochemical CO_2 reduction with enhanced efficiency and durability. *J. Catal.* **392**, 49-55 (2020).

26 Zhong, H. et al. Covalent organic framework hosting metalloporphyrin-based carbon dots for visible-light-driven selective CO_2 reduction. *Adv. Funct. Mater.* **30**, 2002654 (2020).

27 Chen, X. et al. Integrating single Ni sites into biomimetic networks of covalent organic frameworks for selective photoreduction of CO_2. *Chem. Sci.* **11**, 6915-6922 (2020).

28 Yang, S. et al. Microenvironments enabled by covalent organic framework linkages for modulating active metal species in photocatalytic CO_2 reduction. *Adv. Funct. Mater.* **32**, 2110694 (2022).

29 Dong, M. et al. CO_2 dominated bifunctional catalytic sites for efficient industrial exhaust conversion. *Adv. Funct. Mater.* **32**, 2110136 (2022).

30 Fu, Z. et al. A stable covalent organic framework for photocatalytic carbon dioxide reduction. *Chem. Sci.* **11**, 543-550 (2020).

31 Zou, L. et al. Photoelectron transfer mediated by the interfacial electron effects for boosting visible-light-driven CO_2 reduction. *ACS Catal.* **12**, 3550-3557 (2022).

32 Yu, B., et al. Robust biological hydrogen-bonded organic framework with post-functionalized rhenium(I) sites for efficient heterogeneous visible-light-driven CO_2 reduction. *Angew. Chem. Int. Ed.* **60**, 8983-8989 (2021).
